# Skp2 stabilizes Mcl-1 and confers radioresistance in colorectal cancer

**DOI:** 10.1038/s41419-022-04685-0

**Published:** 2022-03-18

**Authors:** Xinfang Yu, Li Zhou, Wenbin Liu, Lijun Liu, Feng Gao, Wei Li, Haidan Liu

**Affiliations:** 1 Department of Cardiovascular Surgery, The Second Xiangya Hospital of Central South University, Changsha, Hunan 410011 People’s Republic of China; 2 Clinical Center for Gene Diagnosis and Therapy, The Second Xiangya Hospital of Central South University, Changsha, Hunan 410011 People’s Republic of China; 3 Department of Medicine, Baylor College of Medicine, Houston, TX 77030 USA; 4 Department of Pathology, National Clinical Research Center for Geriatric Disorders, Xiangya Hospital of Central South University, Changsha, Hunan 410008 People’s Republic of China; 5 Department of Pathology, Hunan Cancer Hospital, Changsha, Hunan 410013 People’s Republic of China; 6 Department of Ultrasonography, The Third Xiangya Hospital of Central South University, Changsha, Hunan 410013 People’s Republic of China; 7 Department of Radiology, The Third Xiangya Hospital of Central South University, Changsha, Hunan 410013 People’s Republic of China

**Keywords:** Biological sciences, Ubiquitylation

## Abstract

Overexpression of Skp2 plays a critical role in tumorigenesis and correlates with poor prognosis in human malignancies. Thus, Skp2 has been proposed as an attractive target for anti-tumor interventions. The expression of Skp2 in human colorectal cancer (CRC) and the role of Skp2 in tumorigenic properties and irradiation sensitivities of CRC cells were examined by anchorage-dependent and -independent growth assays, immunoblot, flow cytometry, immunohistochemical staining, ubiquitination analysis, co-immunoprecipitation assay, CRISPR-Cas9-based gene knockout, and xenograft experiments. Skp2 is highly expressed in CRC patient tissues. Blocking Skp2 expression reduces the tumorigenic properties of CRC cells in vitro and in vivo. Depletion of Skp2 confers sensitivity to irradiation of CRC cells. Skp2 deficiency enhances irradiation-induced intrinsic apoptosis by facilitating E3 ligase FBW7-mediated Mcl-1 ubiquitination and degradation. Knockout of Skp2 sensitizes CRC cells to irradiation treatments in vivo. Our findings indicate that Skp2 stabilizes Mcl-1, and targeting Skp2 in combination with traditional radiotherapy might be efficacious in treating CRC.

## Introduction

Colorectal cancer (CRC) ranks third for incidence and second for leading cause of cancer-related mortality worldwide [1]. Curative-intent surgery combined with adjuvant radiotherapy is a mainstay of therapy for patients with CRC [2]. Nevertheless, CRC patients face challenges related to treatment failure due to inherent and acquired radiation resistance. Novel agents target various genes, such as MEK, VEGF, PKC, HSP90, PARP, PD-1, PD-L1, and CTLA4, developed as radiosensitizers and conducted clinical trials in the treatment of colorectal cancer [2]. To improve the effectiveness of radiotherapy for CRC, discovering potential targets to improve the radiosensitivity of CRC has important clinical significance.

Irradiation (IR) exerts its cytotoxic effects by inducing cell death, such as intrinsic apoptosis, which is regulated by the antiapoptotic and proapoptotic Bcl-2 family members. Disruption of the balance between antiapoptotic and proapoptotic Bcl-2 family members can change the cell fate from survival to apoptosis. Myeloid cell leukemia 1 (Mcl-1) is an antiapoptotic Bcl-2 family member and is one of the most frequently overexpressed proteins in human cancers [3], including lung cancer [4], colorectal [5], liver [6], prostate cancer [7], and multiple myeloma [8]. Mcl-1 inhibits cell death by binding to pro-apoptotic Bcl-2 family members to suppress mitochondrial outer membrane permeabilization and caspase activation, through which tumor cells evade the fate of death. Overexpression of Mcl-1 is an important reason for the resistance to various cancer therapies, including radio- and chemotherapies [9, 10]. Moreover, several studies have demonstrated that downregulating Mcl-1 expression or reducing its stability benefits the treatment of a variety of cancers [11–15]. Therefore, Mcl-1 has emerged as an attractive target for therapeutic strategies.

In the present study, we found that the depletion of Skp2 (S-phase kinase-associated protein 2) enhances irradiation-induced apoptosis, accompanied by the decrease of the Mcl-1 protein level in human colorectal cancer cells. Targeting the Skp2-Mcl-1 axis is a promising anti-tumor strategy to overcome radioresistance in CRC.

## Materials and methods

### Reagents and antibodies

Compounds, including MG132, cycloheximide (CHX) and SB216763 (#S1075), were purchased from Selleck Chemicals (Houston, TX). Chemical reagents, including Tris, NaCl, SDS, and DMSO, were purchased from Sigma-Aldrich for molecular biology and buffer preparation (St. Louis, MO). Cell culture media and supplements were from Invitrogen (Grand Island, NY). Antibodies against Skp2 (#2652, IB:1:2000, IHC: 1:100), α-Tubulin (#2144, IB:1:10000), VDAC1 (#4866, IB:1:2000), Ubiquitin (#3936, IB: 1:1000), Mcl-1(#5453, IB: 1:1000), Cytochrome c (#11940, IB:1:1000), Bax (#5023, IB: 1:1000), GSK3β (#12456, 1:1000), p-GSK3β-Ser9 (#5558, 1:1000), p-Akt-Ser473 (#4060, 1:1000), Akt (#4691, 1:1000), Cleaved-caspase 3 (#9664, IB: 1:2000) and Cleaved-PARP (#5625, IB: 1:2000) were obtained from Cell Signaling Technology, Inc. (Beverly, MA). Antibodies against β-actin (A5316, IB: 1:10000), Flag tag (F3165, IB: 1:10000), and Flag-HRP (A8592, IB: 1:20000) were from Sigma-Aldrich (St. Louis, MO). Antibodies against p-Mcl-1-Ser159 (ab111574, 1:1000), Ki67 (ab16667, IHC: 1: 250), FBW7 (ab109617, IHC: 1:100), FBW7 (ab187815, 1:2000), Mcl-1 (ab32087, IHC:1:100), donkey anti-rabbit IgG H&L (Alexa Fluor®568) (ab175470), and donkey anti-mouse IgG H&L (Alexa Fluor® 488) (ab150105) were purchased from Abcam (Cambridge, UK). Mcl-1 (sc-819) for immunoprecipitate was from Santa Cruz (Dallas, TX). Secondary antibodies, including anti-rabbit IgG HRP (#7074) and anti-mouse IgG HRP (#7076), were purchased from Cell Signaling Technology. Antibody conjugates were visualized by chemiluminescence (ECL; cat#34076, Thermo Fisher Scientific).

### Cell lines and cell culture

Human colorectal cancer cell lines HCT116 and HT29 were purchased from the American Type Culture Collection (ATCC, Manassas, VA). The 293T cell was purchased from ATCC and maintained in DMEM medium supplemented with 10% FBS and 1% antibiotics. All cells were maintained at 37 °C in a humidified incubator with 5% CO_2_ according to the ATCC protocols. The cells were cytogenetically tested and authenticated before being frozen.

### Immunohistochemical staining (IHC)

This study was approved by the Institute Research Ethics Committees of Xiangya Hospital, Central South University. Human colorectal cancer tissues and the paired adjacent tissues were obtained from the Department of Pathology at Xiangya Hospital with written informed consent (*n* = 87). All the patients received no treatment before surgery. The tissues were fixed, embedded, and subjected to IHC analysis as described previously [13]. Briefly, after incubating at 65 °C for 1 h, the tissue slides were submersed into sodium citrate buffer (10 mM, pH6.0) and boiled for 10 min, followed by incubation in 3% H_2_O_2_ for 10 min. Tissue slides were blocked with 50% goat serum albumin at room temperature for 1 h and incubated with the primary antibody in a humidified chamber overnight in a cold room. Tissue slides were washed with PBS and hybridized with the secondary antibody at room temperature for 45 min. Hematoxylin was used for counterstaining. Slides were viewed and photographed under a light microscope and analyzed using the Image-Pro Plus software (version 6.2) program (Media Cybernetics). The immunoreactions were evaluated independently by two pathologists as described previously [16]. Briefly, the percentage of positive cells was scored as follows: 0, no positive cells; 1, ≤10% positive cells; 2, 10–50% positive cells; 3, >50% positive cells. Staining intensity was scored as follows: 0, no staining; 1, weak staining; 2, moderate staining; 3, dark staining. The staining intensity score between 0–1, 1–2, and 2–3 was further scored on a decile scale. Comprehensive score = staining percentage × intensity. Skp2, Mcl-1, or FBW7 expression: ≤2 indicates low expression level; >2 indicates high expression level.

### Transient transfection and generation of silencing stable cell lines

The generation of gene stable silencing cell line was performed as described previously [17]. Two different single-guide RNAs (sgRNAs) were used to generate CRISPR-Cas9-based Skp2 knockout constructs (sgSkp2#1 forward, 5′-AAGACTTTGTGATTGTCCGC-3′, reverse, 5′-GCGGACAATCACAAAGTCTT-3′; sgSkp2#2 forward, 5′-GCAACGTTGCTACTCAGGTC-3′, reverse, 5′-GACCTGAGTAGCAACGTTGC-3′). The Skp2 stable knockout single clone was generated by transient transfection of sgSkp2 plasmids followed by selection with 1 μg/mL puromycin for three weeks. The shGFP (#110318, Addgene) was used as a shCtrl. Mcl-1 stable knockdown cells were generated using shRNA (AAACCCAGGGCTGCCTTGGAAAAG), and selected by 1 mg/mL puromycin for 3 weeks. The Control siRNA (sc-37007), Mcl-1 siRNA (sc-35877), FBW7 siRNA (sc-37547) were purchased from Santa Cruz Biotechnology (Dallas, TX). Cells were grown in 6-well plates and transfected with 100 pmol small interfering RNA oligonucleotide using HiPerFect transfection reagent (Cat. 301705, Qiagen) for 72 h as described previously [18]. Cells were harvested for protein extraction and immunoblotting to confirm Mcl-1 or FBW7 knockdown.

### Protein preparation and Western blotting

Whole-cell lysates were extracted with RIPA buffer (20 mM NAP, pH 7.4, 150 mM NaCl, 1% Triton, 0.5% Sodium-deoxycholate, and 0.1% SDS) supplemented with protease inhibitors. Lysates were sonicated and centrifuged at 12,000 × *g* for 15 min. Protein concentration was determined using the BCA Assay Reagent (#23228, Pierce, Rockford, IL). Western blotting was performed as previously described [19].

### Real-time PCR analysis

The primers for Mcl-1 are as follows: Forward sequence: CCAAGAAAGCTGCATCGAACCAT; Reverse sequence: CAGCACATTCCTGATGCCACCT. The qPCR analysis was performed using the ABI 7900HT with the following PCR program: Stage 1: Activation: 50 °C for 2 min; Stage 2: Pre-soak:95 °C for 10 min; Stage 3: Denaturation: 95 °C for 15 s, annealing: 60 °C for 1 min; Stage 4: Melting curve: 95 °C for 15 s, 60 °C for 15 s, 95 °C for 15 s.

### Cell viability assay

Cells were seeded at a density of 2 × 10^3^ cells per well in 96-well plates in 100 μL of culture medium containing10% FBS and incubated in a 37 °C, 5% CO_2_ incubator. Cells were treated with or without IR (2 Gy). After culturing for another 24, 48, or 72 h, the MTS reagent (#G3581, Promega, Madison, WI) was added to each well, and cells were incubated for another 1 h at 37 °C and measured according to the standard procedures.

### Anchorage-independent cell growth assay

Cells (8 × 10^3^ per well) were treated with or without IR (2 Gy) and seeded into six-well plates with 0.3% Basal Medium Eagle agar containing 10% FBS and cultured. The cultures were maintained at 37 °C in a 5% CO_2_ incubator for two weeks, and colonies were counted under a microscope.

### Plate colony formation assay

Cells were treated with or without IR (2 Gy, X-RAD 320, Precision X-ray, Inc.), and then seeded into the 6 cm plate (5 × 10^2^/well). The cultures were maintained at 37 °C in a 5% CO_2_ incubator for two weeks. The colonies were fixed with 4% paraformaldehyde and stained with 0.5% crystal violet. The numbers of the colony were counted under a microscope. Three independent experiments were performed in triplicate.

### Trypan blue exclusion assay

The cell number and viability were assessed by counting the cells with a hemocytometer (Neubauer Chamber, Germany) using the trypan blue reagent, which distinguishes alive (bright) from dead cells or non-viable cells (blue ones).

### Isolation of mitochondrial fractions

Cells from 10 cm plates were treated with or without IR (2 Gy), harvested, and centrifuged at 850 × *g* for 2 min at 4 °C. The Mitochondria Isolation Kit (#89874, Thermo Fisher Scientific) was used to extract the mitochondrial fractions according to the manufacturers’ standard procedures.

### Flow cytometry

Cells were seeded into six-well plates for 24 h and subjected to IR (2 Gy) treatment. After treatment, attached and floating cells were harvested. The cells were suspended in 1 × 10^6^ cells/mL for apoptosis analysis, and 5 μL Annexin V and Propidium Iodide staining solution was added to 300 μL of the cell suspension. After incubating at room temperature for 15 min at dark, stained cells were assayed and quantified using a FACSort Flow Cytometer (BD, SanJose, CA, USA).

### Ubiquitination assay

The ubiquitination assay was performed as described previously [16]. Briefly, cells were harvested and lysed with modified RIPA buffer (20 mM NAP, pH 7.4, 150 mM NaCl, 1% Triton, 0.5% Sodium-deoxycholate, and 1% SDS) supplemented with protease inhibitors and 10 mM N-Ethylmaleimide (NEM). After sonication, the lysates were boiled at 95 °C for 15 min, diluted with RIPA buffer containing 0.1% SDS, then centrifuged at 4 °C (16,000 × *g* for 15 min). The supernatant was isolated and incubated with specific antibody and protein A/G Sepharose beads overnight at 4 °C. After extensive washing, bound proteins were eluted with 2 × SDS sample loading buffer and separated on an SDS-PAGE, followed by Western blotting analysis.

### In vivo tumor growth assay

The in vivo animal study was approved by the Institutional Animal Care and Use Committee (IACUC) of Central South University (Changsha, China). All mice were maintained and manipulated according to strict guidelines established by the Medical Research Animal Ethics Committee, Central South University, China. Cells (1 × 10^6^) were s.c.injected into the 6-week-old athymic nude mice (*n* = 5) at the right flank to generate the xenograft mouse model. IR treatment was initiated when tumor volume reached 100 mm^3^. Mice were exposed to local ionizing radiation (2 Gy/twice per week, irradiated with X-rays using X-RAD 320, Precision X-ray, Inc.) and tumors were measured by caliper every three days. Tumor volume was recorded and calculated according to the following formula: tumor volume (mm^3^) = (length × width × width/2). Mice were monitored until the endpoint. At that time, mice were euthanized and tumors extracted. Tumor mass was subjected to IHC staining.

### Statistical analysis

Statistical analyses were performed using SPSS (version16.0 for Windows, SPSS Inc, Chicago, IL, USA) and GraphPad Prism 5 (GraphPad 5.0, San Diego, CA, USA). All quantitative data are expressed as mean ± s.e.m of three independent experiments. The Student’s *t*-test or ANOVA evaluated the difference between means. Clinicopathologic significance in clinical samples was assessed by the *χ*^2^ test or Fisher exact test for categorical data. Mann–Whitney *U*-test was used when the data did not fit a normal distribution. The Pearson rank correlation was used for correlation tests. Wilcoxon matched-pairs signed-rank test was used for evaluating the expression level difference between adjacent and tumor. A probability value of *p* < 0.05 was used as the criterion for statistical significance.

## Results

### Skp2 affects the tumorigenic properties and irradiation sensitivities of human colorectal cancer cells

**Fig. 1:**
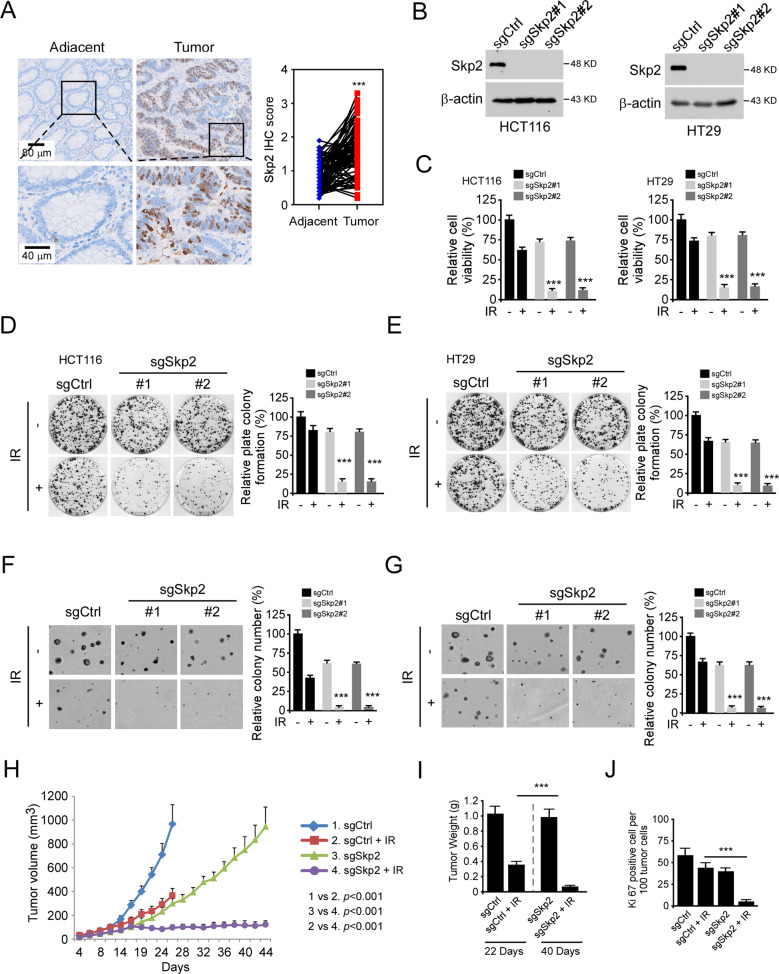
Skp2 is required for the maintaining of tumorigenic properties of human colorectal cancer (CRC) cells. **A** Left, the representative staining images of CRC specimens and adjacent tissues; right, quantification of the staining intensity using Image-Pro PLUS (v.6) and Image J (NIH) computer software. ***p < 0.001. a significant difference between groups as indicated. **B** Immunoblot (IB) analysis of Skp2 protein level in Skp2 knock out CRC stable cell lines. **C** MTS assay was performed to determine the cell viability of Skp2 knock out CRC stable cell lines treated with/without 2 Gy irradiation (IR). ***p < 0.001. **D**, **E** Plate colony formation assay analysis of the colony formation of Skp2 knock out HCT116 (**D**) and HT29 (**E**) stable cell lines treated with/without IR (2 Gy). ***p < 0.001. **F**, **G** Soft agar assay determination of the anchorage-independent cell growth of Skp2 knock out HCT116 (**F**) and HT29 (**G**) stable cell lines treated with/without IR (2 Gy). ***p < 0.001. **H**–**J** Average tumor volume (**H**), average tumor weight (**I**), and the population of Ki67 positive cells (**J**) of HCT116-sgCtrl and HCT116-sgSkp2 xenografts treated with/without IR. ***p < 0.001.

To determine whether Skp2 is related to the tumorigenesis of human colorectal cancer, immunohistochemical (IHC) staining was performed to examine the protein level of Skp2 in colorectal cancer tissues. The results showed that Skp2 was upregulated in CRC tissues compared to the paired adjacent tissues (Fig. 1A). To investigate whether Skp2 affects the sensitivity of human CRC cells to irradiation (IR), we constructed Skp2 stable knockout HCT116 and HT29 cells. We found that depletion of Skp2 (Fig. 1B) significantly decreased cell viability (Fig. 1C) and plate colony formation (Fig. 1D, E) in the presence of irradiation (2 Gy) in both HCT116 and HT29 cells. Anchorage-independency is one of the hallmarks of cancer cells and allows tumor cells to expand and invade adjacent tissues. Our data showed that the anchorage-independent colony formation potential of both Skp2-knockout HCT116 and HT29 cells in soft agar was impaired in the presence of irradiation (Fig. 1F, G). We next constructed a xenograft mouse model using HCT116-sgCtrl and HCT116-sgSkp2 stable cells. Xenograft tumors derived from Skp2-knockout HCT116 cells were treated with irradiation and exhibited a significant decrease in tumor growth, tumor mass, and tumor cell proliferation compared to tumors derived from Skp2-knockout cells that did not receive the irradiation or to tumors retaining Skp2 and treated with irradiation (Fig. 1H–J). These results suggest that blocking Skp2 expression reduces the tumorigenic properties, and Skp2 deficiency confers sensitivity to irradiation of CRC cells.

### Depletion of Skp2 enhances IR-induced apoptosis in human colorectal cancer cells

**Fig. 2:**
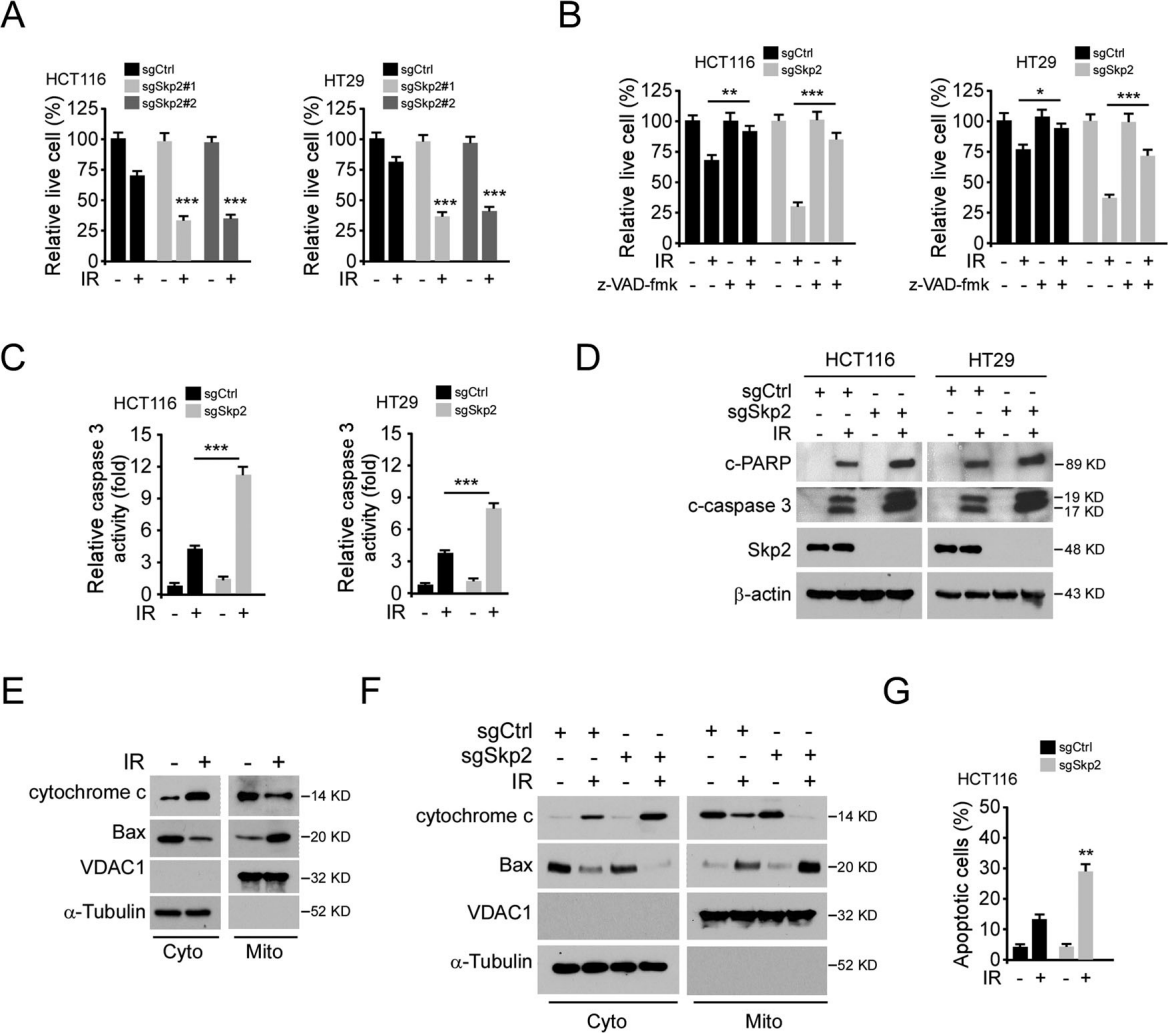
Depletion of Skp2 enhances IR-induced intrinsic apoptosis. **A** Skp2 knockout CRC stable cells were treated with/without IR (2 Gy) and cultured for 72 h, live cell population was determined by trypan blue exclusion assay. ***p < 0.001. **B**, **C** Skp2 knockout CRC stable cells were pretreated with pan-caspase inhibitor z-VAD-fmk for 4 h, followed by 2 Gy IR treatment. Cells were cultured for 72 h, live cell population was determined by trypan blue exclusion assay (**B**), caspase 3 activity was examined by Caspase 3 Assay Kit (**C**). *p < 0.05, **p < 0.01, ***p < 0.001. **D** Skp2 knockout HCT116 (left) and HT29 (right) stable cells were treated with/without IR (2 Gy) and cultured for 72 h, whole-cell extract (WCE) was subjected to IB analysis. **E** HCT116 cells were treated with/without IR (2 Gy) and cultured for 72 h, subcellular fractions were isolated and subjected to IB analysis. **F**, **G** Skp2 knockout HCT116 stable cells were treated with/without IR (2 Gy) and cultured for 72 h, subcellular fractions were isolated and subjected to IB analysis (**F**), the population of apoptotic cells was examined by flow cytometry (**G**). **p < 0.01.

We next determined whether Skp2 deficiency affects irradiation**-**induced apoptosis of human CRC cells. The trypan blue exclusion assay showed that knockout of Skp2 decreased the population of live cells in the presence of irradiation in both HCT116 and HT29 cells (Fig. 2A). Pretreated with apoptosis inhibitor z-VAD-fmk partially recovered the population of live cells in the presence of irradiation (Fig. 2B), indicating that apoptosis is involved. By analyzing the activity of caspase 3, we showed that depletion of Skp2 increased the activity of caspase 3 in the presence of irradiation in both HCT116 and HT29 cells (Fig. 2C). Furthermore, the IB data demonstrated that IR-induced cleaved-caspase 3 and -PARP were upregulated robustly in Skp2 knockout HCT116 and HT29 stable cells (Fig. 2D). The subcellular fractions, including mitochondrial and cytosolic fractions, were isolated to determine whether intrinsic apoptosis was involved. Irradiation decreased the expression of Bax in the cytosolic fraction but enhanced its protein level in the mitochondrial fraction of HCT116 cells (Fig. 2E). The release of cytochrome c from mitochondria to the cytoplasm was consistently increased with irradiation treatment (Fig. 2E). Knockout of Skp2 with irradiation treatment further promoted the protein level of cytochrome C in the cytosolic fraction, whereas the expression of cytochrome c in the mitochondrial fraction was reduced. Moreover, knockout of Skp2 with irradiation further increased the presence of Bax on mitochondria and decreased it in the cytoplasm (Fig. 2F) in HCT116 cells. Flow cytometry results indicated that knockout of Skp2 increased the apoptotic cells triggered by irradiation (Fig. 2G). These data suggest that depletion of Skp2 enhances irradiation-induced intrinsic apoptosis in human colorectal cancer cells.

### Irradiation decreases Mcl-1 protein level in Skp2 deficient colorectal cancer cells

**Fig. 3:**
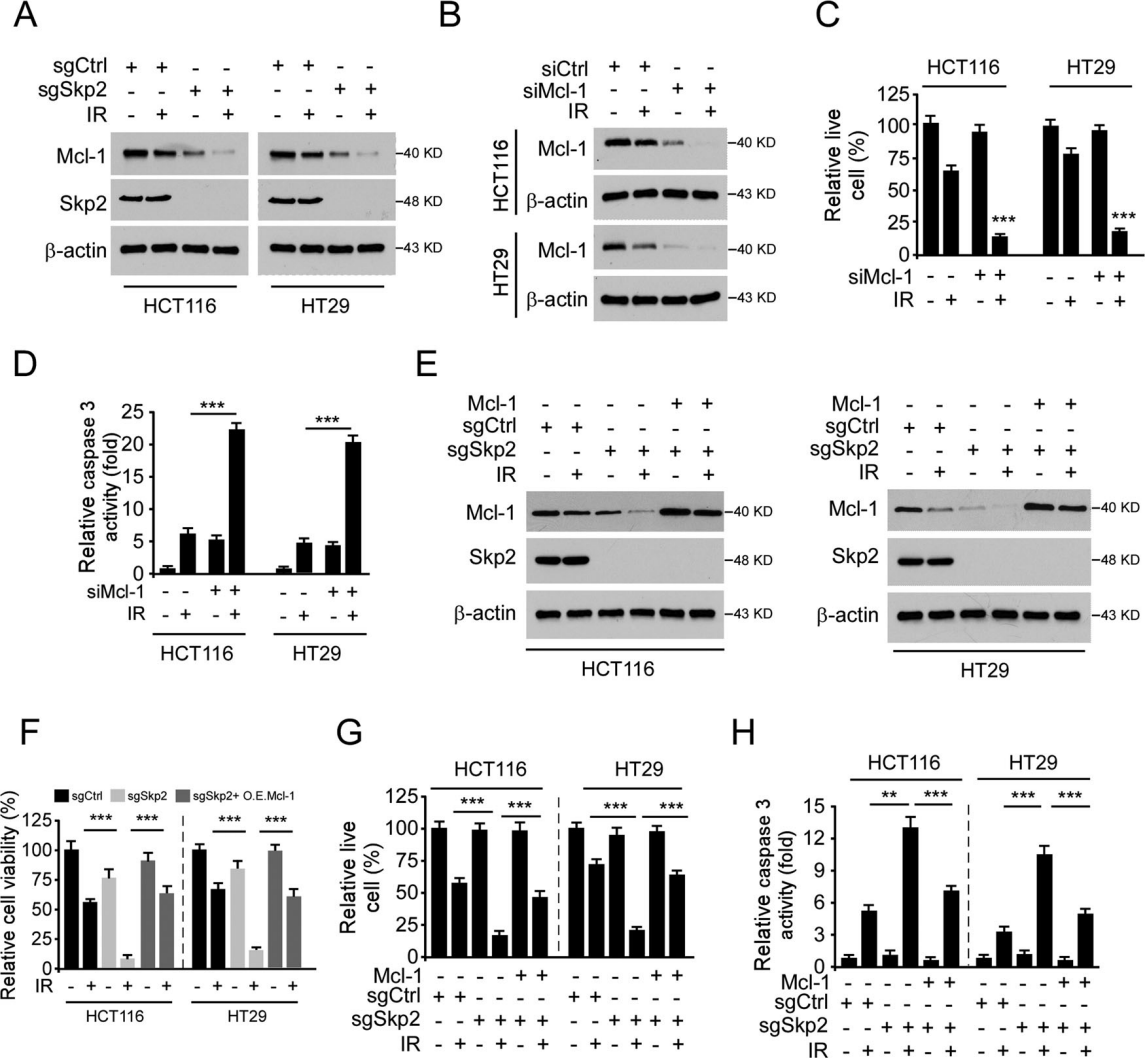
Irradiation decreases Mcl-1 protein level. **A** Skp2 knockout CRC stable cells were treated with/without IR (2 Gy) and cultured for 72 h, WCE was subjected to IB analysis. **B**–**D** HCT116, and HT29 cells were transfected with siMcl-1 for 24 h, followed by IR (2 Gy) treated and cultured for 72 h. WCE was subjected to IB analysis (**B**). The live cell population was determined by trypan blue exclusion assay (**C**). Caspase 3 activity was examined by Caspase 3 Assay Kit (**D**). ***p < 0.001. **E**–**H** Mcl-1 was transiently transfected into Skp2 depleted HCT116 and HT29 cells using lipofectamine 2000 and maintained for 24 h. Cells were treated by IR (2 Gy) and cultured for 72 h. WCE was subjected to IB analysis (**E**). Cell viability and live cell population were determined by MTS assay (**F**) and trypan blue exclusion assay (**G**), respectively. Caspase 3 activity was examined by Caspase 3 Assay Kit (**H**). **p < 0.01, ***p < 0.001.

To determine the mechanisms of how depletion of Skp2 promoted IR-induced apoptosis, we examined the anti-apoptotic Bcl-2 family members in Skp2 knockout stable cells. The results showed that depletion of Skp2 downregulated Mcl-1 expression, but not that of Bcl-2 or Bcl-xL (Supplementary Fig. 1A). Moreover, the protein level of Mcl-1 was further decreased when Skp2 depletion was combined with irradiation treatment in both HCT116 and HT29 cells (Fig. 3A). Knockdown of Mcl-1 (Fig. 3B) and exposure to irradiation dramatically decreased the population of live cells (Fig. 3C), increased the activity of caspase 3 (Fig. 3D) in Mcl-1 silenced HCT116 and HT29 cells. We next determined whether overexpression of Mcl-1 compromised irradiation-induced apoptosis. The results revealed that ectopic overexpression of Mcl-1 (Fig. 3E) compromised irradiation decreased cell viability (Fig. 3F), anchorage-dependent (Supplementary Fig. 1B) and -independent colony formation (Supplementary Fig. 1C), as well as live cell population (Fig. 3G) in Skp2 depleted HCT116 and HT29 cells. Consistently, the activity of caspase 3 was reduced with Mcl-1 transfection (Fig. 3H). Furthermore, reintroduction of Skp2 in Skp2-null HCT116 cells rescued Mcl-1 expression and compromised IR-induced caspase 3 activations (Supplementary Fig. 1D, E). These data suggest that irradiation decreases the Mcl-1 protein level in Skp2 deficient colorectal cancer cells and contributes to irradiation-induced intrinsic apoptosis.

### Irradiation promotes Mcl-1 ubiquitination and degradation

**Fig. 4:**
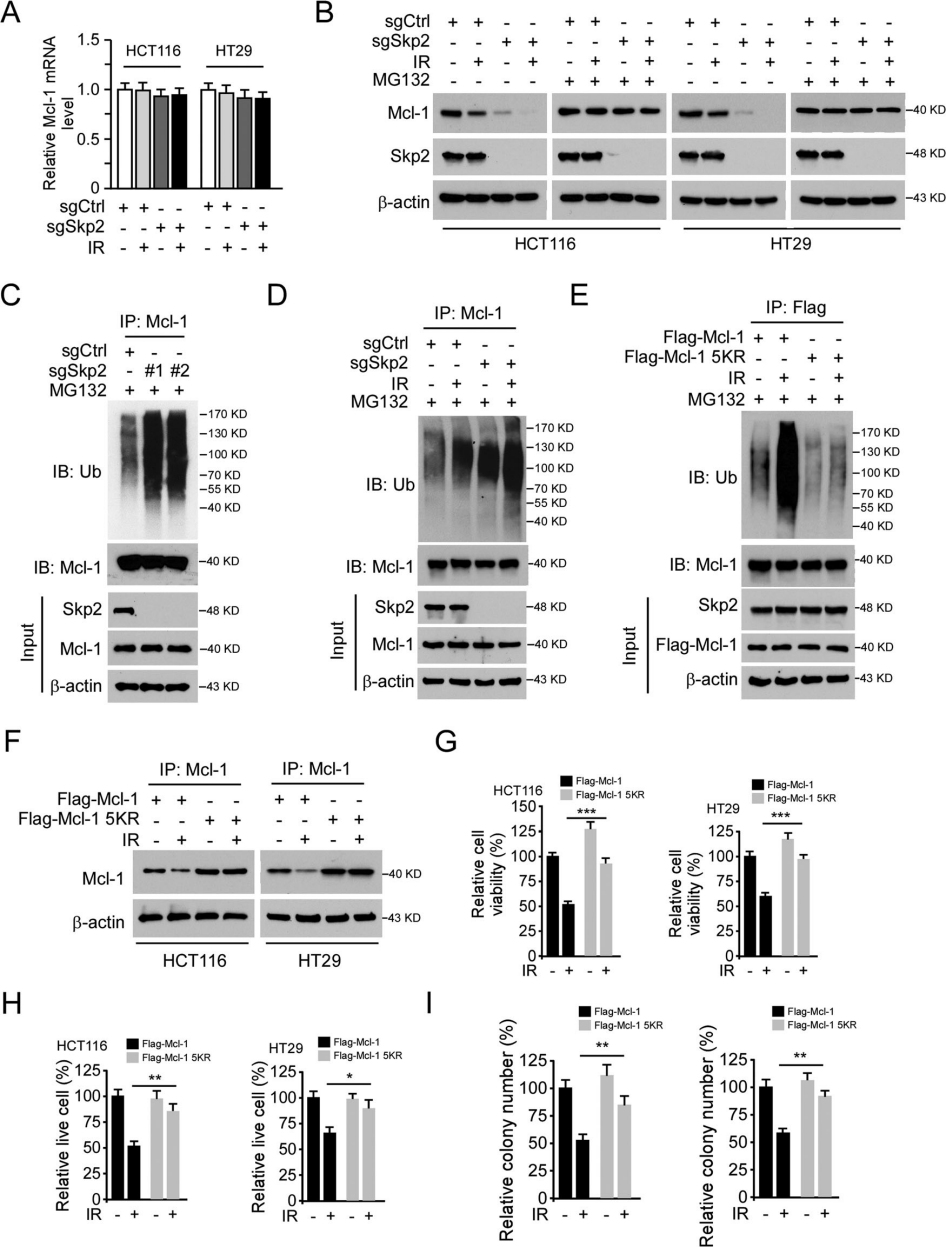
Irradiation promotes Mcl-1 ubiquitination and degradation. **A** Mcl-1 mRNA expression in Skp2 depleted CRC cells with IR treatment was examined by real-time RT-PCR. **B** Skp2 depleted HCT116, and HT29 cells were treated with IR (2 Gy) and cultured for 72 h, MG132 (25 μM) was added to the cell culture medium and maintained for 6 h. WCE was subjected to IB analysis. **C** Skp2 knockout HCT116 cells were treated with MG132 for 6 h, WCE was prepared and subjected to Mcl-1 ubiquitination analysis. **D** Skp2 depleted HCT116 cells were treated with MG132 (25 μM) for 6 h, followed by IR (2 Gy) treatment and cultured for 1 h. WCE was subjected to Mcl-1 ubiquitination analysis. **E** Flag-Mcl-1 wild type or 5KR mutant was transfected into HCT116 cells using lipofectamine 2000 for 48 h and treated with IR as indicated. WCE was extracted after IR treatment for 1 h and subjected to Mcl-1 ubiquitination analysis. **F** Flag-Mcl-1 wild type or 5KR mutant was transfected into HCT116 cells for 24 h, followed by IR (2 Gy) treatment, and cultured for 72 h. WCE was subjected to IB analysis. **G**–**I** Flag-Mcl-1 wild type or 5KR mutant was transfected into HCT116 and HT29 cells for 24 h, followed by IR (2 Gy) treatment and cultured for 72 h. Cell viability and live cell population were determined by MTS assay (**G**) and trypan blue exclusion assay (**H**), respectively. Colony formation was examined by plate colony formation assay (**I**). *p < 0.05, **p < 0.01, ***p < 0.001.

To determine the mechanism of how irradiation downregulates Mcl-1 expression, we first performed qRT-PCR to analyze the transcription of Mcl-1 after Skp2 depletion with or without irradiation exposure. The result showed that the mRNA level of Mcl-1 was unaffected in Skp2 depleted CRC cells with or without irradiation treatment (Fig. 4A), strongly suggesting that post-translational mechanisms chiefly regulate Mcl-1. The Western blot results showed that Mcl-1 protein levels were reduced following Skp2 depletion and further decreased upon irradiation treatment in HCT116 and HT29 cells (Fig. 4B). Notably, the proteasome inhibitor, MG132, restored Mcl-1 expression, suggesting that the degradation of Mcl-1 was enhanced in Skp2-null CRC cells (Fig. 4B). The ubiquitination analysis revealed that depletion of Skp2 increased Mcl-1 ubiquitination in HCT116 cells, indicating that Skp2 is required for Mcl-1 stabilization (Fig. 4C). Moreover, Mcl-1 ubiquitination was increased by irradiation treatment, which was strongly promoted in Skp2 depleted HCT116 cells (Fig. 4D). These data suggest that irradiation decreased Mcl-1 expression is related to protein degradation. Human Mcl-1 protein contains a total of 13 lysine residues, and five lysine residues, including K5, K40, K136, K194, and K197, have been shown to be ubiquitinated by FBW7 [20]. To determine whether IR-induced Mcl-1 ubiquitination occurs on these lysine sites, we constructed a 5KR mutant, in which all of these five lysine residues were mutated to arginine. The in vivo ubiquitination result showed that IR-induced Mcl-1 ubiquitination was reduced markedly in the Mcl-1 5KR mutant in HCT116 cells (Fig. 4E). IR decreased the protein level of Mcl-1 WT but not 5KR mutant in both HCT116 and HT29 cells (Fig. 4F). Consistently, ectopic overexpression of Mcl-1 5KR rescued IR-decreased cell viability (Fig. 4G), live cell population (Fig. 4H), and colony formation (Fig. 4I). These results suggest that irradiation promotes Mcl-1 ubiquitination and degradation, depletion of Skp2 enhanced irradiation-induced Mcl-1 destruction.

### FBW7 is required for IR-induced Mcl-1 ubiquitination

To determine how IR-induced Mcl-1 ubiquitination in Skp2 knockout cells, we first examined which signaling pathway was changed in these stable cells. Previous studies have shown that the E3 ligase Skp2 plays a crucial role in tumorigenesis and Herceptin sensitivity in breast cancer by activating Akt signaling in an Akt K63-linked ubiquitination-dependent manner [21]. Indeed, we found that depletion of Skp2 reduced K63-linked ubiquitination and phosphorylation of Akt (Supplementary Fig. 2A). Moreover, the immunoblotting results confirmed that IR treatment further reduced Akt activity and downstream kinase GSK3β, in Skp2 knockout HCT116 and HT29 cells (Supplementary Fig. 2B, C), indicating that Skp2 is required for Akt signaling activation in CRC cells even with IR treatment. Overexpression of constitutively activated Akt1 (Myr-Akt) impaired IR-induced Mcl-1 reduction and cleaved-caspase 3 induction (Supplementary Fig. 2D). GSK3β-mediated Mcl-1 S159 phosphorylation promoted for Mcl-1 ubiquitination [22]. To determine whether IR decreased Mcl-1 is dependent on GSK3β, we generated GSK3β knockdown HCT116 cells and found that silencing of GSK3β by shRNA restored Mcl-1 protein level and compromised IR-induced cleaved-caspase 3 expression (Supplementary Fig. 2E). Importantly, blocking the kinase activity of GSK3β by small molecule inhibitor SB216763 restored Mcl-1 expression in Skp2-null HCT116 cells (Supplementary Fig. 2F). Our data support the notion that IR reduces Mcl-1 in CRC cells is dependent on Skp2-Akt-GSK3β signaling.

**Fig. 5:**
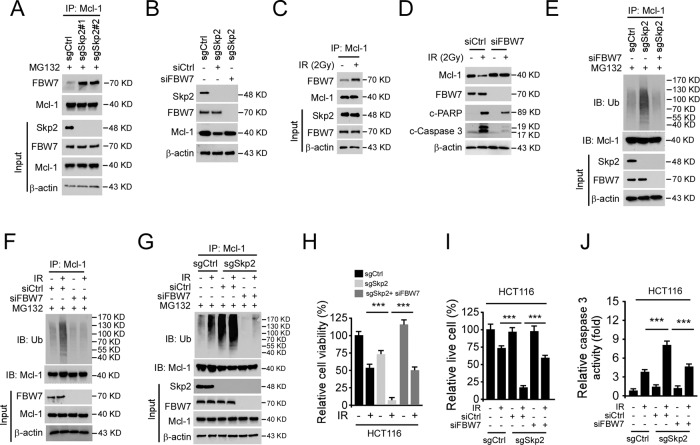
FBW7 is required for IR-induced Mcl-1 ubiquitination. **A** Skp2 knockout HCT116 cells were treated with MG132 for 6 h, WCE was subjected to co-immunoprecipitation (Co-IP) analysis. **B** siFBW7 was transfected into Skp2 depleted HCT116 cells and subjected to IB analysis. **C** HCT116 cells were treated with IR (2 Gy), WCE was collected 1 h later and subjected to Co-IP analysis. **D** siFBW7 was transfected into HCT116 cells for 24 h, followed by IR (2 Gy) treatment. Cells were cultured for 72 h, WCE was subjected to IB analysis. **E** siFBW7 was transfected into Skp2 depleted HCT116 cells for 48 h, followed by MG132 treatment for 6 h, WCE was subjected to IP-mediated Mcl-1 ubiquitination analysis. **F** HCT116 cells were transfected with siCtrl or siFBW7 and cultured for 48 h. After being incubated with MG132 for 6 h, the cells were treated with IR. WCE was collected 1 h later and subjected to IP-mediated Mcl-1 ubiquitination analysis. **G**–**J** Skp2 depleted HCT116 cells were transfected with siCtrl or siFBW7 and cultured for 48 h. After being incubated with MG132 for 6 h, the cells were treated with IR. WCE was collected 1 h later and subjected to IP-mediated Mcl-1 ubiquitination analysis (**G**). Cell viability and live cell population was determined by MTS assay (**H**) and trypan blue exclusion assay (**I**), respectively. Caspase 3 activity was examined by Caspase 3 Assay Kit (**J**). ***p < 0.001.

Because GSK3β-induced Mcl-1 phosphorylation enhanced FBW7-mediated Mcl-1 degradation and FBW7 is frequently mutated in around 15–20% of human CRC, we next examined the interaction between Mcl-1 and the E3 ligase FBW7 in CRC cells. The result showed that FBW7 is bound with Mcl-1 in HCT116 cells, and this interaction was elevated by the depletion of Skp2 (Fig. 5A). Knockdown of FBW7 rescued Mcl-1 expression in Skp2-knockout HCT116 (Fig. 5B) and HT29 (Supplementary Fig. 3A) cells. Co-IP results demonstrated that irradiation (2 Gy) significantly increased the interaction between Mcl-1 and FBW7 in HCT116 (Fig. 5C) and HT29 (Supplementary Fig. 3B) cells. Moreover, knockdown of FBW7 compromised irradiation-induced reduction of Mcl-1 and impaired IR-induced apoptosis in HCT116 (Fig. 5D) and HT29 (Supplementary Fig. 3C) cells. We further examined whether FBW7 regulates Mcl-1 ubiquitination. The result showed that suppression of FBW7 by small interfering RNA compromised Mcl-1 ubiquitination in Skp2 depletion cells (Fig. 5E). Consistently, knockdown of FBW7 also decreased IR-induced Mcl-1 ubiquitination in HCT116 cells (Fig. 5F). To determine whether IR-induced Mcl-1 ubiquitination in Skp2-null cell is dependent on FBW7, we silenced FBW7 and examined Mcl-1 ubiquitination. The result showed that IR strongly induced Mcl-1 ubiquitination in Skp2-null HCT116 cells, and the knockdown of FBW7 compromised this efficacy (Fig. 5G). Consistently, knockdown of FBW7 attenuated IR-decreased cell viability (Fig. 5H) and live cell population (Fig. 5I). Moreover, depletion of FBW7 decreased IR-induced caspase 3 activity (Fig. 5J) in Skp2-null HCT116 cells. These results suggest that FBW7 plays a critical role in IR-promoted Mcl-1 ubiquitination and destruction.

### Irradiation inhibits in vivo tumor growth

**Fig. 6:**
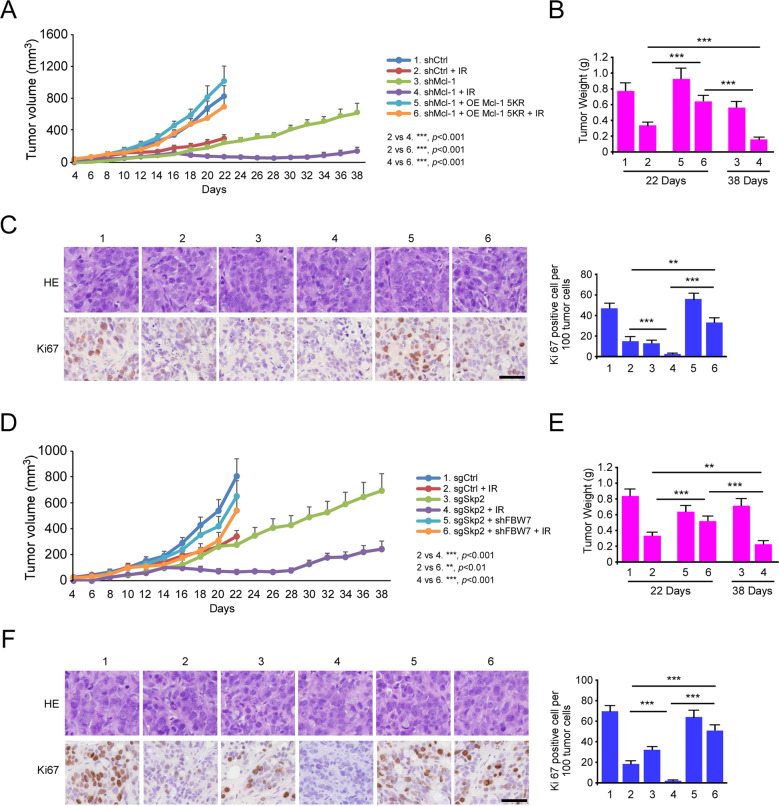
Irradiation inhibits in vivo tumor growth. **A**–**C** Mcl-1 5KR reintroduction into Mcl-1 knockdown HCT116 cells rescues tumorigenesis under IR treatment. Mcl-1 5KR mutant was reintroduced into Mcl-1 knockdown HCT116 cells by lipofectamine 2000. The positive colonies were selected by G418 for 3 weeks, followed by injection into nude mice to establish the xenograft mouse model. Tumor size was monitored (**A**). Tumors were weighed (**B**). Ki67 positive cells were examined by IHC staining (**C**). Scale bar, 50 µm. **p < 0.01, ***p < 0.001. **D**–**F** Knockdown of FBW7 in Skp2-null HCT116 cells rescues tumorigenesis under IR treatment. FBW7 was silenced in Skp2-null HCT116 cells and injected into nude mice to establish the xenograft mouse model. Tumor size was monitored (**D**). Tumors were weighed (**E**). Ki67 positive cells were examined by IHC staining (**F**). Scale bar, 50 µm. **p < 0.01, ***p < 0.001.

We next investigated whether Mcl-1 affects the sensitivity of CRC cells to radiotherapy in vivo. We performed the xenograft tumors using HCT116 cells. Xenograft tumors derived from Mcl-1 knockdown HCT116 cells were treated with irradiation and exhibited a significant decrease in tumor growth (Fig. 6A), tumor mass (Fig. 6B), and tumor cell proliferation (Fig. 6C) compared to tumors derived from Mcl-1 knockdown cells that did not receive the irradiation treatment or to tumors retaining WT Mcl-1 and treated with irradiation (Fig. 6A). In the Mcl-1 knockdown HCT116 tumors, reintroduction of Mcl-1 5KR mutant impaired the anti-tumor effectiveness of irradiation treatment (Fig. 6A–C). We next determined the radiotherapeutic function of Mcl-1 ubiquitination in vivo. Xenograft tumors derived from Skp2-null HCT116 cells that were treated with irradiation exhibited reduced tumor growth (Fig. 6D), tumor mass (Fig. 6E), and tumor cell proliferation (Fig. 6F). However, silent of FBW7 in Skp2-null HCT116 cells rescues tumorigenesis under irradiation treatment (Fig. 6D–F). These results suggest that Mcl-1 stabilization confers radioresistance in CRC cells. Knockout of Skp2 sensitized CRC cells to radiotherapy is dependent on FBW7-mediated Mcl-1 ubiquitination and degradation.

### Skp2 positively correlates with Mcl-1 in CRC tissues

**Fig. 7:**
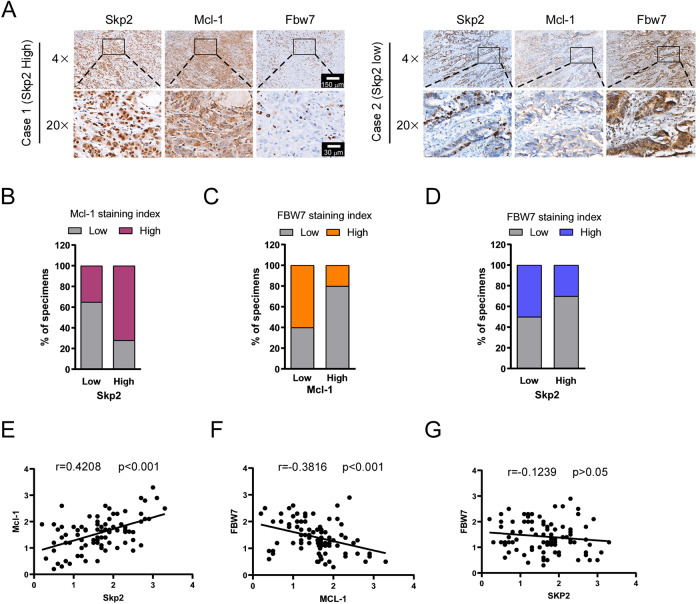
Skp2 positively correlates with Mcl-1 in CRC tissues. **A** representative cases from 87 CRC specimens were analyzed by IHC staining with Skp2, Mcl-1, and FBW7. **B** The percentage of samples displaying low or high Skp2 expression compared to the expression levels of Mcl-1. **C** The percentage of specimens displaying low or high Mcl-1 expression compared to the expression levels of FBW7. **D** The percentage of specimens displaying low or high Skp2 expression compared to the expression levels of FBW7. **E** Scatterplot showing the positive correlation between Skp2 and Mcl-1. **F** Scatterplot showing the negative correlation between Mcl-1 and FBW7. **G** Scatterplot showing no correlation between Skp2 and FBW7.

To determine the clinical relevance of our findings, we evaluated Skp2, Mcl-1, and FBW7 protein levels in 87 primary CRC specimens (Supplementary Tables 1 and 2) by immunohistochemical (IHC) analysis. The results showed that Skp2 is significantly more highly expressed in CRC tumor tissue than in adjacent non-tumor tissues (Supplementary Table 1). Moreover, a high Skp2 protein level is significantly associated with a more advanced tumor stage, as well as lymph node involvement (Supplementary Table 2). The representative staining images with a high or low level of Skp2 expression, as well as Mcl-1 and FBW7, were shown (Fig. 7A). By systematically analyzing the IHC staining results, the details were summarized according to the score of Skp2, Mcl-1, and FWB7. Among 87 patients, 38 cases of the high level of Mcl-1 were seen in all 53 individuals with a high level of Skp2 staining (Fig. 7B). In comparison, 39 of 49 patients with a high level of Mcl-1 exhibited downregulated protein level of FBW7 (Fig. 7C). Also, high Skp2 expression was accompanied by a low level of FBW7, 37 cases of the low level of FBW7 were detected in all 53 patients with a high level of Skp2 (Fig. 7D). As expected, a statistically significant positive correlation between Skp2 and Mcl-1 and a negative correlation between FBW7 and Mcl-1 (Fig. 7E, F) were observed. However, it appears that Skp2 and FBW7 were not significantly negatively correlated in our CRC cohort (Fig. 7G). These findings suggest that Skp2 positively correlates with Mcl-1, which is negatively correlated with E3 ligase FBW7, which may contribute to tumorigenesis of CRC.

## Discussion

Mcl-1 is frequently overexpressed in human tumors and contributes to tumor development, progression, and poor prognosis. Mcl-1 is a relatively short-lived protein and post-translationally regulated through ubiquitination and deubiquitination. E3 ubiquitin ligases, including Mule [23], β-TrCP [22], and FBW7 [13, 20, 24] have been identified to directly interact and induce Mcl-1 polyubiquitination and proteasomal degradation in response to apoptotic stimuli. E3 ligase Parkin has been implicated in Mcl-1 degradation in response to mitochondrial depolarization [25, 26]. Trim17-mediated ubiquitination and degradation of Mcl-1 initiate apoptosis in neurons [27]. FBXO4 is recently identified as an E3 ubiquitin ligase to interact and promote Mcl-1 ubiquitination and degradation in lung cancer [12]. APC/C^Cdc20^ has been shown to engage in the ubiquitination of Mcl-1 and to control Mcl-1 stability during mitosis [15, 28]. Intriguingly, E3 ligase TRAF6 promotes nondegradative K63-linked polyubiquitination of Mcl-1 that antagonizes Mcl-1 interaction with the 20S proteasome, thereby protecting Mcl-1 from degradation elicited by chemotherapeutic drugs [29]. Deubiquitinases can reverse ubiquitylation. Deubiquitinases USP9X [30], Ku70 [31], JOSD1 [32], DUB3/USP17L2 [33], and USP13 [34] have been described to stabilize Mcl-1, promote tumor cell survival and suppress apoptosis. Our results showed that knockout of Skp2 did not cause the accumulation of Mcl-1 protein (Fig. 3A, lane 1 vs. lane 3, in both left and right panels), and depletion of Skp2 increased Mcl-1 ubiquitination (Fig. 4C), indicating that IR-induced Mcl-1 reduction is likely regulated through other E3 ligases rather than Skp2. We subsequently confirmed that another F-box protein, FBW7, fulfills this function. Since the regulation of Mcl-1 stabilization depends on different stimuli and cell types, the identification of more E3 ligases and DUBs that regulate Mcl-1 is important for Mcl-1-targeted therapies.

Regulation of Mcl-1 ubiquitination is primarily mediated by its phosphorylation at multiple sites in response to different signaling events [35]. Multiple Mcl-1 phosphorylation sites have been identified, including Ser64, Thr92, Ser121, Ser159, and Thr163 [36], which slows or accelerates Mcl-1 degradation [37]. Among them, phosphorylation on Thr92 and Thr163 by Cdk2/cyclinE [38], Ser159 by GSK3β [39], Thr92 by Cdk5 [40], and Thr92 by CDK1/cyclin B1 [28] led to increased ubiquitinylation and degradation of Mcl-1. Furthermore, a previous study indicated that Mcl-1 phosphorylation is required for UV-stimulated Mcl-1 degradation [41]. Our current data showed that IR treatment reduced Akt activity and downstream kinase GSK3β in Skp2 knockout HCT116 and HT29 cells (Supplementary Fig. 2B, C). Blocking the kinase activity of GSK3β by small molecule inhibitor SB216763 restored Mcl-1 expression in Skp2-null HCT116 cells (Supplementary Fig. 2F). Our results suggested that phosphorylation of Mcl-1 on Ser159 is essential for IR-induced Mcl-1 downregulation, and GSK3β mediates this Mcl-1 phosphorylation in response to IR treatment. In addition, we found that both depletion of Skp2 (Fig. 5A) and IR treatment (Fig. 5C) significantly increased the FBW7 and Mcl-1 interaction, thus promoting FBW7**-**mediated phosphorylated-Mcl-1 degradation and ultimately sensitizing CRC cells to radiotherapy. However, we could not exclude the possibility that Mcl-1 is phosphorylated on other sites by other kinases to regulate its stabilization, and the mechanisms underlying how Skp2 regulates FBW7 and Mcl-1 interaction need to be further explored.

Skp2 and FBW7 are two different recognition subunits of the Skp1-Cullin1-F-box protein (SCF) E3 ligase complex that recognize specific substrates for ubiquitination [42] and also have opposite functional and biological effects. Skp2 functions as an oncoprotein and exerts oncogenic functions through ubiquitination of its substrates such as p21 [43], p27 [44], p57 [45], E-cadherin [46], FOXO1 [47], Akt [48], and others. Therefore, Skp2 plays a crucial role in governing many critical cellular processes, including cell growth, apoptosis, differentiation, cell cycle progression, migration, invasion, and metastasis [49]. Skp2 is frequently overexpressed in various human cancer [49, 50], including colorectal cancer [17]. Moreover, overexpression of Skp2 is positively correlated with TNM stage, node capsular invasion, lymphovascular invasion and is strongly associated with poor prognosis in CRC patients [51, 52]. Additionally, Skp2 has been reportedly involved in developing drug resistance [53–55] or radiation resistance [56]. On the contrary, FBW7 functions as a tumor suppressor and is commonly downregulated in cancer. Aberration or inactivation of FBW7 expression has been observed in human cancers, such as breast cancer [57], and leukemia [58], which is thought to be involved in tumorigenesis, progress, prognosis, and drug resistance [24, 59, 60]. FBW7 mRNA expression is reported to be significantly reduced in colorectal cancer [20, 61, 62]. FBW7 targets many well-characterized oncoproteins, including c-MYC [63], CyclinE [64], c-JUN [65], Mcl-1 [24], and Notch intracellular domain 1 (NICD1) [66] for ubiquitylation-mediated proteasomal degradation [59]. Intriguingly, the stability and proteasomal degradation of FBW7 itself is regulated by deubiquitinase USP9X [67] and E3 ligase TRIP12 [68]. We found that depletion of Skp2 significantly increased the FBW7 and Mcl-1 interaction (Fig. 5A), enhanced IR-induced Mcl-1 ubiquitination (Fig. 4D), which was dependent on the E3 ligase FBW7 (Fig. 5E), and ultimately sensitizing CRC cells to radiotherapy both in vitro (Fig. 1) and in vivo (Fig. 6). Though the precise mechanism underlying Skp2 deficiency-increased FBW7 and Mcl-1 interaction remains unclear, the oncoprotein Skp2 is still an attractive anti-cancer target. Our results showed a trend that Skp2 is required to maintain Mcl-1 stability in IR-treated CRC cells, and depletion of Skp2 increased Mcl-1 ubiquitination (Fig. 4C), and Skp2 is positively correlated with Mcl-1 expression in CRC tissues (Fig. 7H), suggesting Skp2 plays a role in maintaining of Mcl-1 stability and expression upregulation, finally contributes to tumorigenesis of CRC. It has been reported that a growing number of E3 ligases function as transcriptional coactivators. For example, Skp2 has been revealed to act as a transcriptional coactivator with c-Myc to regulate gene expression [69, 70]. The simultaneous presence of c-Myc and Skp2 were detected at the c-Myc target gene promoter [70]. Whether Skp2 is associated with other transcriptional factor and/or other proteins and recruited to the *Mcl-1* promoter to regulate Mcl-1 transcription and expression require further exploration.

An increasing number of pharmacological agents targeted for inhibiting E3 ligases and deubiquitinases have been developed. The MDM2 inhibitor Nutlin-3 effectively restores p53 function and induces cell cycle arrest and apoptosis in MDM2 expression human rhabdomyosarcoma cells with wild-type p53 [71]. Serdemetan, an MDM2 inhibitor, mitigates experimental pulmonary hypertension (PH) in mice, partially through the inhibition of MDM2-mediated ubiquitination of angiotensin-converting enzyme 2 (ACE2) and thus rectified ACE2 expression [72]. APC/C^cdc20^ inhibitor Apcin suppresses the metastasis in triple-negative breast cancer [73]. Skp2 inhibitor SZL P1–41 restricts cancer stem cell traits and cancer progression by selectively suppressing Skp2 ubiquitin ligase activity [74]. USP14 inhibitor IU1 significantly increases CD36 ubiquitination and stabilizes CD36 protein by removing the polyubiquitin chains, decreasing foam cell formation by downregulating CD36-mediated lipid uptake, and providing a potential therapeutic target for atherosclerosis [75]. Spautin-1 reportedly suppresses autophagy by inhibiting the USP10 and USP13 deubiquitinases [76]. P22077, a dual inhibitor of USP7/USP47 [77], overcomes tyrosine kinase inhibitor resistance and eradicates leukemia stem/progenitor cells in chronic myelogenous leukemia through inhibiting its novel substrate Y-box binding protein 1 (YB-1) deubiquitination and suppresses DNA damage repair [78]. Several pharmacological agents have been shown to diminish Mcl-1 expression by inhibiting Mcl-1 production or enhancing Mcl-1 degradation. For instance, the USP9X inhibitor WP1130 lowers Mcl-1 levels in chronic myelogenous leukemia and enhances sensitivity to apoptosis by facilitating Mcl-1 degradation [79]. Thus, the further discovery of highly selective and effective E3 ligases and deubiquitinases inhibitors with fewer side effects are required for clinical treatment.

Targeting Mcl-1 appears to be a promising strategy in cancer therapy. Unfortunately, there were no Mcl-1 inhibitors have been approved currently. Our results indicated that irradiation induces CRC cell apoptosis through FWB7-mediated Mcl-1 degradation. Mcl-1 regulates the radiosensitivity of CRC cells and might be a target for CRC radiosensitization. Combining E3 ligases upstream of Mcl-1 with traditional radiotherapy would be efficacious in treating CRC.

## Supplementary information

Combined Supplementary Information

Checklist

## Data Availability

All data and materials supporting the conclusions of this study have been included within the article and the supplemental data. The uncropped WB blot results were included in the Supplementary Information file.

## References

[1] Sung H, Ferlay J, Siegel RL, Laversanne M, Soerjomataram I, Jemal A (2021). Global Cancer Statistics 2020: GLOBOCAN estimates of incidence and mortality worldwide for 36 cancers in 185 countries. CA Cancer J Clin.

[2] George TJ, Franke AJ, Chakravarthy AB, Das P, Dasari A, El-Rayes BF (2019). National Cancer Institute (NCI) state of the science: Targeted radiosensitizers in colorectal cancer. Cancer..

[3] Beroukhim R, Mermel CH, Porter D, Wei G, Raychaudhuri S, Donovan J (2010). The landscape of somatic copy-number alteration across human cancers. Nature.

[4] Nangia V, Siddiqui FM, Caenepeel S, Timonina D, Bilton SJ, Phan N (2018). Exploiting MCL1 dependency with combination MEK + MCL1 inhibitors leads to induction of apoptosis and tumor regression in KRAS-mutant non-small cell lung cancer. Cancer Discov..

[5] Greaves G, Milani M, Butterworth M, Carter RJ, Byrne DP, Eyers PA (2019). BH3-only proteins are dispensable for apoptosis induced by pharmacological inhibition of both MCL-1 and BCL-XL. Cell Death Differ.

[6] Song P, Yang S, Hua H, Zhang H, Kong Q, Wang J (2019). The regulatory protein GADD34 inhibits TRAIL-induced apoptosis via TRAF6/ERK-dependent stabilization of myeloid cell leukemia 1 in liver cancer cells. J Biol Chem.

[7] Pradhan AK, Bhoopathi P, Talukdar S, Shen XN, Emdad L, Das SK (2018). Recombinant MDA-7/IL24 suppresses prostate cancer bone metastasis through downregulation of the Akt/Mcl-1 pathway. Mol Cancer Ther.

[8] Siu KT, Huang C, Panaroni C, Mukaihara K, Fulzele K, Soucy R (2019). BCL2 blockade overcomes MCL1 resistance in multiple myeloma. Leukemia..

[9] Kelly PN, Strasser A (2011). The role of Bcl-2 and its pro-survival relatives in tumourigenesis and cancer therapy. Cell Death Differ.

[10] Perciavalle RM, Opferman JT (2013). Delving deeper: MCL-1’s contributions to normal and cancer biology. Trends Cell Biol.

[11] Zhang H, Guttikonda S, Roberts L, Uziel T, Semizarov D, Elmore SW (2011). Mcl-1 is critical for survival in a subgroup of non-small-cell lung cancer cell lines. Oncogene..

[12] Feng C, Yang F, Wang J (2017). FBXO4 inhibits lung cancer cell survival by targeting Mcl-1 for degradation. Cancer Gene Ther.

[13] Gao F, Yu X, Li M, Zhou L, Liu W, Li W (2020). Deguelin suppresses non-small cell lung cancer by inhibiting EGFR signaling and promoting GSK3beta/FBW7-mediated Mcl-1 destabilization. Cell Death Dis.

[14] Wang R, Xia L, Gabrilove J, Waxman S, Jing Y (2016). Sorafenib inhibition of Mcl-1 accelerates ATRA-induced apoptosis in differentiation-responsive AML cells. Clin Cancer Res.

[15] Allan LA, Skowyra A, Rogers KI, Zeller D, Clarke PR. Atypical APC/C-dependent degradation of Mcl-1 provides an apoptotic timer during mitotic arrest. EMBO J. 2018;37:e96831. 10.15252/embj.201796831 PMC6120658 29987118

[16] Yu X, Wang R, Zhang Y, Zhou L, Wang W, Liu H (2019). Skp2-mediated ubiquitination and mitochondrial localization of Akt drive tumor growth and chemoresistance to cisplatin. Oncogene..

[17] Zhou L, Yu X, Li M, Gong G, Liu W, Li T (2020). Cdh1-mediated Skp2 degradation by dioscin reprogrammes aerobic glycolysis and inhibits colorectal cancer cells growth. EBioMedicine..

[18] Liu H, Liu K, Huang Z, Park CM, Thimmegowda NR, Jang JH (2013). A chrysin derivative suppresses skin cancer growth by inhibiting cyclin-dependent kinases. J Biol Chem.

[19] Liu W, Li W, Liu H, Yu X (2019). Xanthohumol inhibits colorectal cancer cells via downregulation of Hexokinases II-mediated glycolysis. Int J Biol Sci.

[20] Inuzuka H, Shaik S, Onoyama I, Gao D, Tseng A, Maser RS (2011). SCF(FBW7) regulates cellular apoptosis by targeting MCL1 for ubiquitylation and destruction. Nature..

[21] Chan CH, Li CF, Yang WL, Gao Y, Lee SW, Feng Z (2012). The Skp2-SCF E3 ligase regulates Akt ubiquitination, glycolysis, herceptin sensitivity, and tumorigenesis. Cell..

[22] Ding Q, He X, Hsu JM, Xia W, Chen CT, Li LY (2007). Degradation of Mcl-1 by beta-TrCP mediates glycogen synthase kinase 3-induced tumor suppression and chemosensitization. Mol Cell Biol.

[23] Zhong Q, Gao W, Du F, Wang X (2005). Mule/ARF-BP1, a BH3-only E3 ubiquitin ligase, catalyzes the polyubiquitination of Mcl-1 and regulates apoptosis. Cell..

[24] Wertz IE, Kusam S, Lam C, Okamoto T, Sandoval W, Anderson DJ (2011). Sensitivity to antitubulin chemotherapeutics is regulated by MCL1 and FBW7. Nature..

[25] Sarraf SA, Raman M, Guarani-Pereira V, Sowa ME, Huttlin EL, Gygi SP (2013). Landscape of the PARKIN-dependent ubiquitylome in response to mitochondrial depolarization. Nature..

[26] Carroll RG, Hollville E, Martin SJ (2014). Parkin sensitizes toward apoptosis induced by mitochondrial depolarization through promoting degradation of Mcl-1. Cell Rep.

[27] Magiera MM, Mora S, Mojsa B, Robbins I, Lassot I, Desagher S (2013). Trim17-mediated ubiquitination and degradation of Mcl-1 initiate apoptosis in neurons. Cell Death Differ.

[28] Harley ME, Allan LA, Sanderson HS, Clarke PR (2010). Phosphorylation of Mcl-1 by CDK1-cyclin B1 initiates its Cdc20-dependent destruction during mitotic arrest. EMBO J.

[29] Choi YB, Harhaj EW (2014). HTLV-1 tax stabilizes MCL-1 via TRAF6-dependent K63-linked polyubiquitination to promote cell survival and transformation. PLoS Pathog.

[30] Schwickart M, Huang X, Lill JR, Liu J, Ferrando R, French DM (2010). Deubiquitinase USP9X stabilizes MCL1 and promotes tumour cell survival. Nature..

[31] Wang B, Xie M, Li R, Owonikoko TK, Ramalingam SS, Khuri FR (2014). Role of Ku70 in deubiquitination of Mcl-1 and suppression of apoptosis. Cell Death Differ.

[32] Wu X, Luo Q, Zhao P, Chang W, Wang Y, Shu T (2020). JOSD1 inhibits mitochondrial apoptotic signalling to drive acquired chemoresistance in gynaecological cancer by stabilizing MCL1. Cell Death Differ.

[33] Wu X, Luo Q, Zhao P, Chang W, Wang Y, Shu T (2019). MGMT-activated DUB3 stabilizes MCL1 and drives chemoresistance in ovarian cancer. Proc Natl Acad Sci USA.

[34] Zhang S, Zhang M, Jing Y, Yin X, Ma P, Zhang Z (2018). Deubiquitinase USP13 dictates MCL1 stability and sensitivity to BH3 mimetic inhibitors. Nat Commun.

[35] Ertel F, Nguyen M, Roulston A, Shore GC (2013). Programming cancer cells for high expression levels of Mcl1. EMBO Rep.

[36] Gores GJ, Kaufmann SH (2012). Selectively targeting Mcl-1 for the treatment of acute myelogenous leukemia and solid tumors. Genes Dev.

[37] Kobayashi S, Lee SH, Meng XW, Mott JL, Bronk SF, Werneburg NW (2007). Serine 64 phosphorylation enhances the antiapoptotic function of Mcl-1. J Biol Chem.

[38] Choudhary GS, Tat TT, Misra S, Hill BT, Smith MR, Almasan A (2015). Cyclin E/Cdk2-dependent phosphorylation of Mcl-1 determines its stability and cellular sensitivity to BH3 mimetics. Oncotarget..

[39] Maurer U, Charvet C, Wagman AS, Dejardin E, Green DR (2006). Glycogen synthase kinase-3 regulates mitochondrial outer membrane permeabilization and apoptosis by destabilization of MCL-1. Mol Cell.

[40] Nikhil K, Shah K (2017). The Cdk5-Mcl-1 axis promotes mitochondrial dysfunction and neurodegeneration in a model of Alzheimer’s disease. J Cell Sci.

[41] Morel C, Carlson SM, White FM, Davis RJ (2009). Mcl-1 integrates the opposing actions of signaling pathways that mediate survival and apoptosis. Mol Cell Biol.

[42] Frescas D, Pagano M (2008). Deregulated proteolysis by the F-box proteins SKP2 and beta-TrCP: Tipping the scales of cancer. Nat Rev Cancer.

[43] Yu ZK, Gervais JL, Zhang H (1998). Human CUL-1 associates with the SKP1/SKP2 complex and regulates p21(CIP1/WAF1) and cyclin D proteins. Proc Natl Acad Sci USA.

[44] Tsvetkov LM, Yeh KH, Lee SJ, Sun H, Zhang H (1999). p27(Kip1) ubiquitination and degradation is regulated by the SCF(Skp2) complex through phosphorylated Thr187 in p27. Curr Biol.

[45] Kamura T, Hara T, Kotoshiba S, Yada M, Ishida N, Imaki H (2003). Degradation of p57Kip2 mediated by SCFSkp2-dependent ubiquitylation. Proc Natl Acad Sci USA.

[46] Inuzuka H, Gao D, Finley LW, Yang W, Wan L, Fukushima H (2012). Acetylation-dependent regulation of Skp2 function. Cell..

[47] Huang H, Regan KM, Wang F, Wang D, Smith DI, van Deursen JM (2005). Skp2 inhibits FOXO1 in tumor suppression through ubiquitin-mediated degradation. Proc Natl Acad Sci USA.

[48] Chan CH, Li CF, Yang WL, Gao Y, Lee SW, Feng Z (2012). The Skp2-SCF E3 ligase regulates Akt ubiquitination, glycolysis, herceptin sensitivity, and tumorigenesis. Cell..

[49] Wang Z, Gao D, Fukushima H, Inuzuka H, Liu P, Wan L (2012). Skp2: a novel potential therapeutic target for prostate cancer. Biochim Biophys Acta.

[50] Wang Z, Liu P, Inuzuka H, Wei W (2014). Roles of F-box proteins in cancer. Nat Rev Cancer.

[51] Shapira M, Ben-Izhak O, Linn S, Futerman B, Minkov I, Hershko DD (2005). The prognostic impact of the ubiquitin ligase subunits Skp2 and Cks1 in colorectal carcinoma. Cancer..

[52] Vasile Bochis O, Achimas-Cadariu P, Vlad C, Fetica B, Corneliu Leucuta D, Ioan (2017). The prognostic role of Skp2 and the tumor suppressor protein p27 in colorectal cancer. J BUON.

[53] Totary-Jain H, Sanoudou D, Dautriche CN, Schneller H, Zambrana L, Marks AR (2012). Rapamycin resistance is linked to defective regulation of Skp2. Cancer Res.

[54] Davidovich S, Ben-Izhak O, Shapira M, Futerman B, Hershko DD (2008). Over-expression of Skp2 is associated with resistance to preoperative doxorubicin-based chemotherapy in primary breast cancer. Breast Cancer Res.

[55] Yang Q, Huang J, Wu Q, Cai Y, Zhu L, Lu X (2014). Acquisition of epithelial-mesenchymal transition is associated with Skp2 expression in paclitaxel-resistant breast cancer cells. Br J Cancer.

[56] Wang XC, Tian LL, Tian J, Jiang XY (2012). Overexpression of SKP2 promotes the radiation resistance of esophageal squamous cell carcinoma. Radiat Res.

[57] Akhoondi S, Lindstrom L, Widschwendter M, Corcoran M, Bergh J, Spruck C (2010). Inactivation of FBXW7/hCDC4-beta expression by promoter hypermethylation is associated with favorable prognosis in primary breast cancer. Breast Cancer Res.

[58] O’Neil J, Look AT (2007). Mechanisms of transcription factor deregulation in lymphoid cell transformation. Oncogene..

[59] Davis RJ, Welcker M, Clurman BE (2014). Tumor suppression by the Fbw7 ubiquitin ligase: Mechanisms and opportunities. Cancer Cell.

[60] Wang Z, Fukushima H, Gao D, Inuzuka H, Wan L, Lau AW (2011). The two faces of FBW7 in cancer drug resistance. Bioessays..

[61] Iwatsuki M, Mimori K, Ishii H, Yokobori T, Takatsuno Y, Sato T (2010). Loss of FBXW7, a cell cycle regulating gene, in colorectal cancer: clinical significance. Int J Cancer.

[62] Yeh CH, Bellon M, Nicot C (2018). FBXW7: A critical tumor suppressor of human cancers. Mol Cancer.

[63] King B, Trimarchi T, Reavie L, Xu L, Mullenders J, Ntziachristos P (2013). The ubiquitin ligase FBXW7 modulates leukemia-initiating cell activity by regulating MYC stability. Cell..

[64] Koepp DM, Schaefer LK, Ye X, Keyomarsi K, Chu C, Harper JW (2001). Phosphorylation-dependent ubiquitination of cyclin E by the SCFFbw7 ubiquitin ligase. Science..

[65] Nateri AS, Riera-Sans L, Da Costa C, Behrens A (2004). The ubiquitin ligase SCFFbw7 antagonizes apoptotic JNK signaling. Science..

[66] Onoyama I, Suzuki A, Matsumoto A, Tomita K, Katagiri H, Oike Y (2011). Fbxw7 regulates lipid metabolism and cell fate decisions in the mouse liver. J Clin Invest.

[67] Khan OM, Carvalho J, Spencer-Dene B, Mitter R, Frith D, Snijders AP (2018). The deubiquitinase USP9X regulates FBW7 stability and suppresses colorectal cancer. J Clin Invest.

[68] Khan OM, Almagro J, Nelson JK, Horswell S, Encheva V, Keyan KS (2021). Proteasomal degradation of the tumour suppressor FBW7 requires branched ubiquitylation by TRIP12. Nat Commun.

[69] Kim SY, Herbst A, Tworkowski KA, Salghetti SE, Tansey WP (2003). Skp2 regulates Myc protein stability and activity. Mol Cell.

[70] von der Lehr N, Johansson S, Wu S, Bahram F, Castell A, Cetinkaya C (2003). The F-box protein Skp2 participates in c-Myc proteosomal degradation and acts as a cofactor for c-Myc-regulated transcription. Mol Cell.

[71] Miyachi M, Kakazu N, Yagyu S, Katsumi Y, Tsubai-Shimizu S, Kikuchi K (2009). Restoration of p53 pathway by nutlin-3 induces cell cycle arrest and apoptosis in human rhabdomyosarcoma cells. Clin Cancer Res.

[72] Shen H, Zhang J, Wang C, Jain PP, Xiong M, Shi X (2020). MDM2-mediated ubiquitination of angiotensin-converting enzyme 2 contributes to the development of pulmonary arterial hypertension. Circulation..

[73] Song C, Lowe VJ, Lee S. Inhibition of Cdc20 suppresses the metastasis in triple negative breast cancer (TNBC). Breast Cancer. 2021;28:1073–1086. 10.1007/s12282-021-01242-z 33813687

[74] Chan CH, Morrow JK, Li CF, Gao Y, Jin G, Moten A (2013). Pharmacological inactivation of Skp2 SCF ubiquitin ligase restricts cancer stem cell traits and cancer progression. Cell..

[75] Zhang F, Xia X, Chai R, Xu R, Xu Q, Liu M (2020). Inhibition of USP14 suppresses the formation of foam cell by promoting CD36 degradation. J Cell Mol Med.

[76] Liu J, Xia H, Kim M, Xu L, Li Y, Zhang L (2011). Beclin1 controls the levels of p53 by regulating the deubiquitination activity of USP10 and USP13. Cell.

[77] Weinstock J, Wu J, Cao P, Kingsbury WD, McDermott JL, Kodrasov MP (2012). Selective dual inhibitors of the cancer-related deubiquitylating proteases USP7 and USP47. ACS Med Chem Lett.

[78] Lei H, Xu HZ, Shan HZ, Liu M, Lu Y, Fang ZX (2021). Targeting USP47 overcomes tyrosine kinase inhibitor resistance and eradicates leukemia stem/progenitor cells in chronic myelogenous leukemia. Nat Commun.

[79] Sun H, Kapuria V, Peterson LF, Fang D, Bornmann WG, Bartholomeusz G (2011). Bcr-Abl ubiquitination and Usp9x inhibition block kinase signaling and promote CML cell apoptosis. Blood..

