# Supplementary material for: Skp2 stabilizes Mcl-1 and confers radioresistance in colorectal cancer
Source: Cell Death Dis. 2022 Mar 18;13(3):249. doi: 10.1038/s41419-022-04685-0 (PMC8930992; doi:10.1038/s41419-022-04685-0)

Supplementary legends

Supplementary Figure 1. Ectopic overexpression of Mcl-1 compromised irradiation decreased anchorage-dependent and -independent colony formation. A, The whole-cell extracts from HCT116 and HT29 cells expression sgCtrl or sgSkp2 stable cells were subjected to IB analysis. B and C, Mcl-1 was transiently transfected into Skp2 depleted HCT116 and HT29 cells, followed by IR (2Gy) treated and cultured for 72 h. Plate colony formation assay analysis of the colony formation (B) and soft agar assays (C) determination of the anchorage-independent cell growth (B) were performed. ***p<0.001. D and E, Skp2 was transiently transfected into Skp2 depleted HCT116 cells using lipofectamine 2000 and maintained for 24 h. Cells were treated by IR (2Gy) and cultured for 72 h. WCE was subjected to IB analysis (D). Caspase 3 activity was examined by Caspase 3 Assay Kit (E). ***p<0.001.

Supplementary Figure 2. Skp2-Akt-GSK3β axis is required for IR-induced Mcl-1 degradation. A, Skp2 knockout HCT116 cells were treated with MG132 for 6 h, WCE was prepared and subjected to Akt ubiquitination analysis using K63-linkage specific polyubiquitin antibody. B and C, Skp2 knockout HCT116 (B) and HT29 (C) stable cells were treated with/without IR (2Gy) and cultured for 72 h, WCE was subjected to IB analysis. D, HCT116 cells were transfected with the Myr-Akt1 construct, followed by treated with IR (2Gy) and cultured for 72 h. WCE was collected and subjected to IB analysis. E, HCT116 cells stable expression shGFP or shGSK3β were treated with IR (2Gy) and cultured for 72 h, WCE were collected and subjected to IB analysis as indicated. F, HCT116 sgSkp2 stable cells were treated with IR (2Gy) and cultured for 48 h, SB216763 was then added to the culture medium and maintained for 24 h, WCE were collected and subjected to IB analysis as indicated.

Supplementary Figure 3. FBW7 is required for IR-induced Mcl-1 ubiquitination. A, siFBW7 was transfected into Skp2 depleted HT29 cells and subjected to IB analysis. B, HT29 cells were treated with IR (2Gy), WCE was collected 1 h later and subjected to Co-IP analysis. C, siFBW7 was transfected into HT29 cells for 24 h, followed by IR (2Gy) treatment. Cells were cultured for 72 h, WCE was subjected to IB analysis.

Supplementary Table 1. Protein expression of Skp2 in CRC tissues and adjacent non-tumor tissues.

Supplementary Table 2. Relationships between the expression of Skp2 and clinical-pathological characteristics in 87 patients with CRC

Supplementary Figure 1

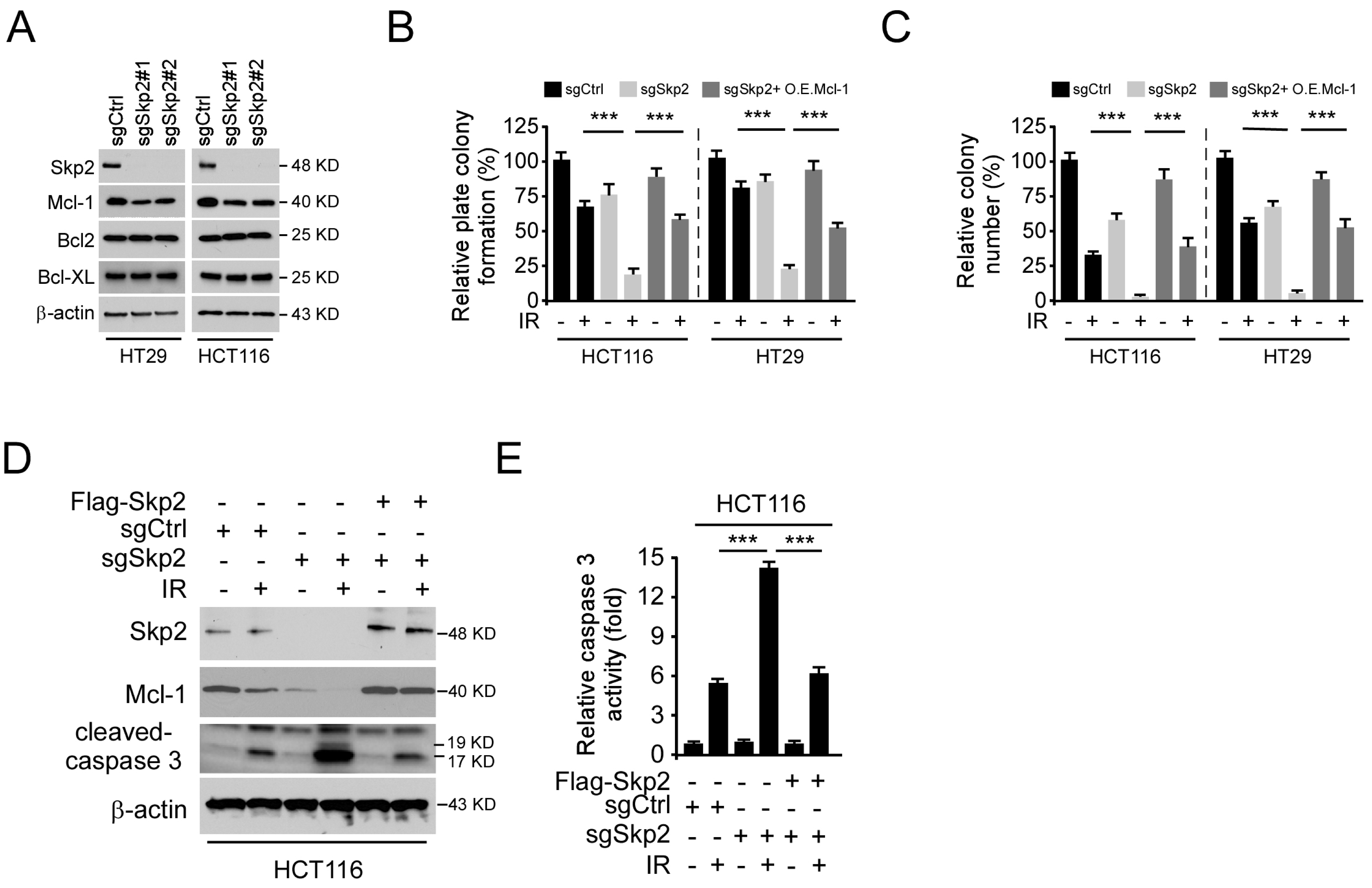

Supplementary Figure 2

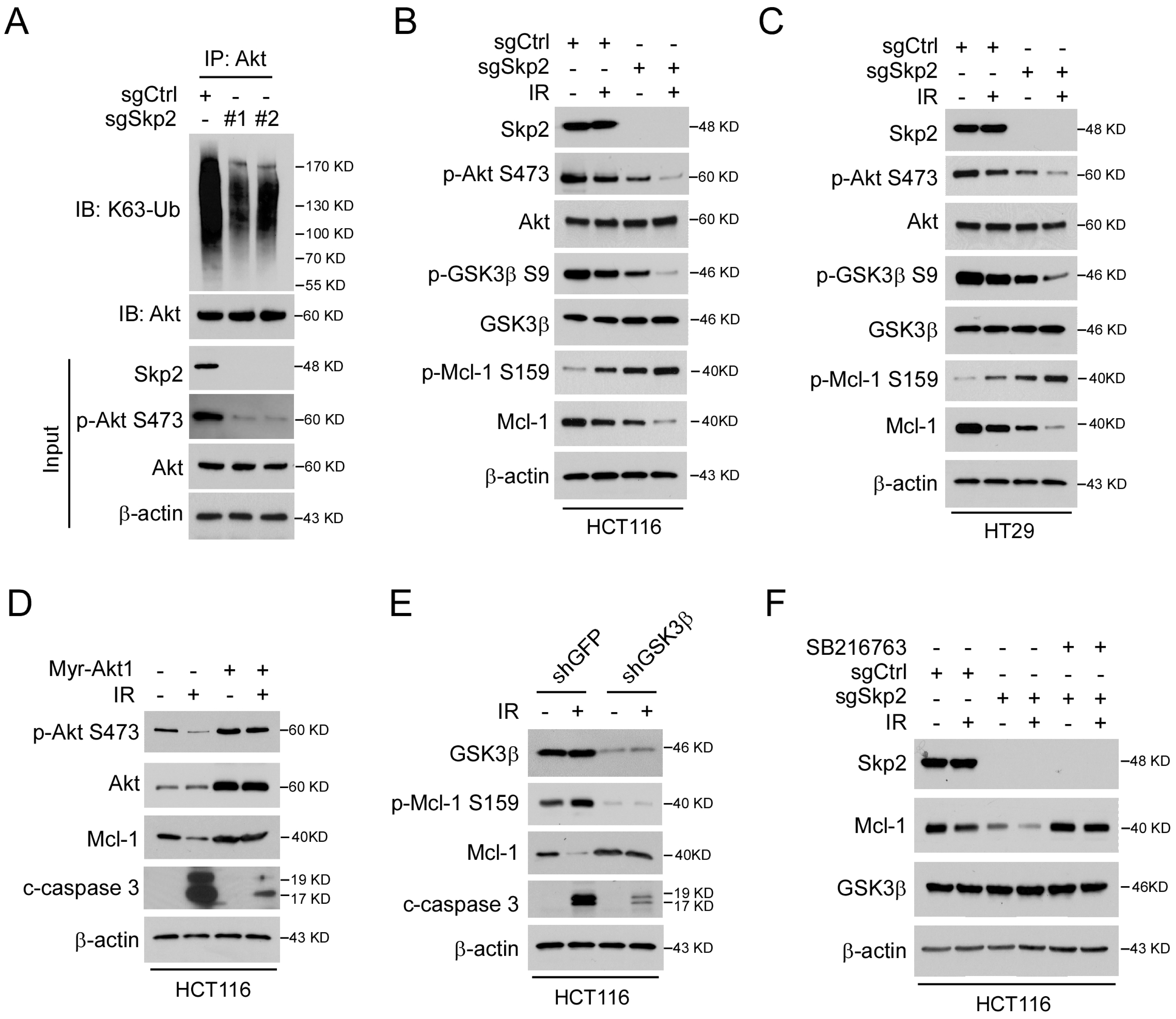

Supplementary Figure 3

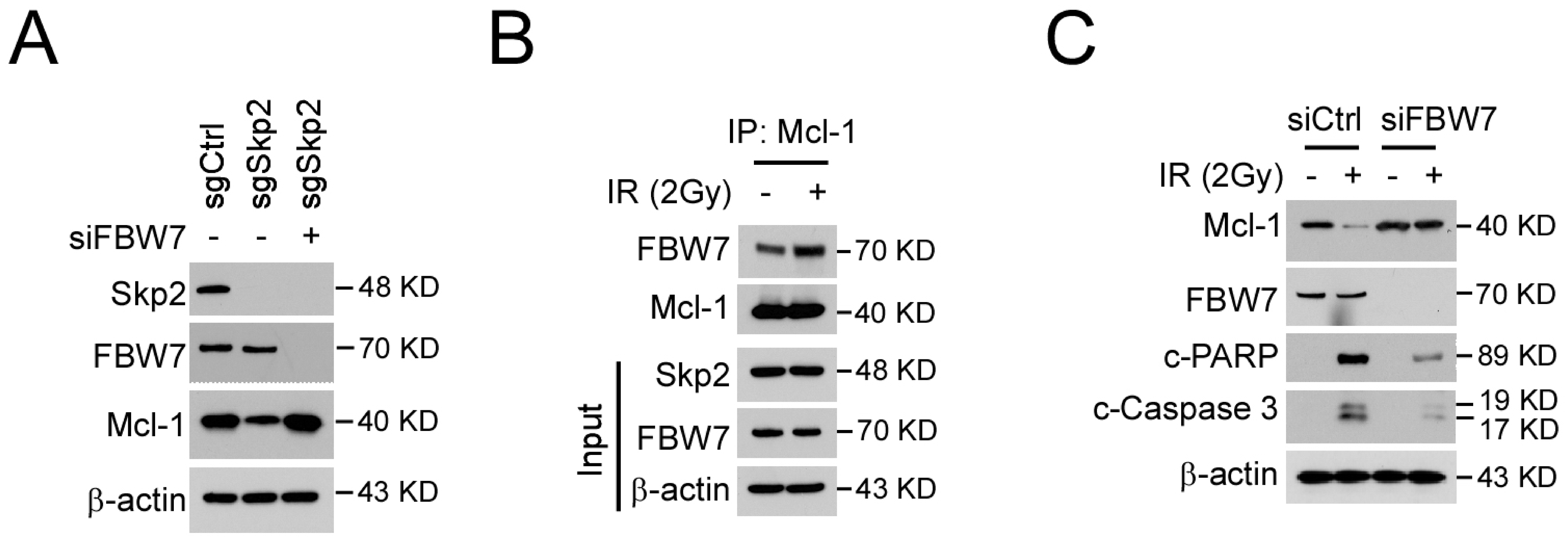

Supplementary Table 1: Protein expression of Skp2 in CRC tissues and adjacent non-tumor tissues.

**Tissue sample**	**No. of patients**	**Skp2**		***p*- value**
		**Low**	**High**	
Tumor	87	34	53	<0.001*
Adjacent	87	82	5	

Chi-square test.

*p<0.05 indicates a significant association among the variables.

Supplementary Table 2: Relationships between the expression of Skp2 and clinical pathological characteristics in 87 patients with CRC.

Characteristics	All cases	Expression of Skp2		*p* -value
		Low(n=34)	High(n=53)	
**Gender**				0.897
Male	53	21	32	
Female	34	13	21	
**Age(years)**				0.761
≤60	35	13	22	
＞60	52	21	31	
**Initial clinical stage**				
≤Ⅱa	39	23	16	0.298
＞Ⅱa	48	11	37	
**Tumor stages**				0.0006*
T1+T2	35	16	19	
T3+T4	52	18	34	
**Lymph nodes status**				0.0000*
N0(negative)	45	27	18	
N1 or above(positive)	42	7	35	

Chi-square test.

*p<0.05 indicates a significant association among the variables.

Uncropped WB data

For Figure 1 B

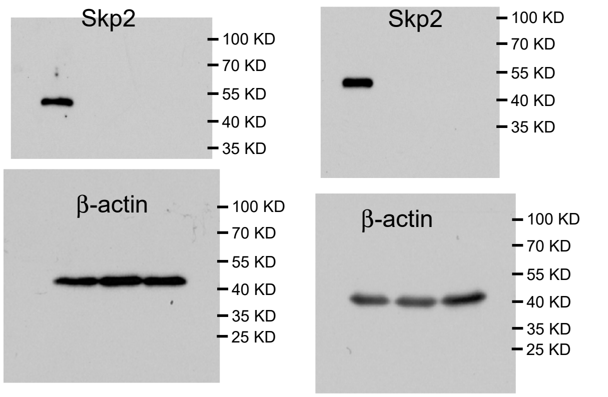

For Figure 2 D

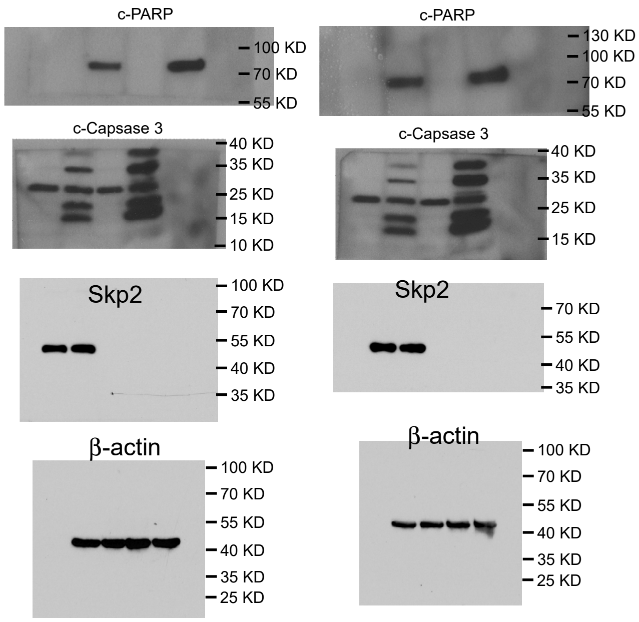

For Figure 2 E

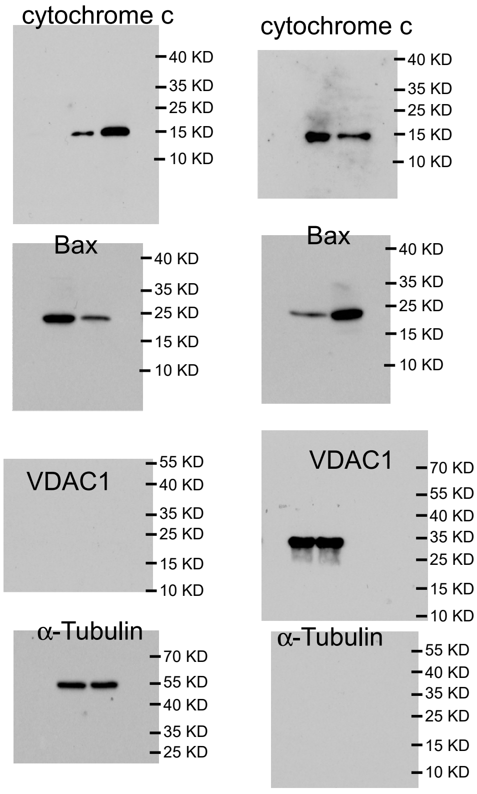

For Figure 2 F

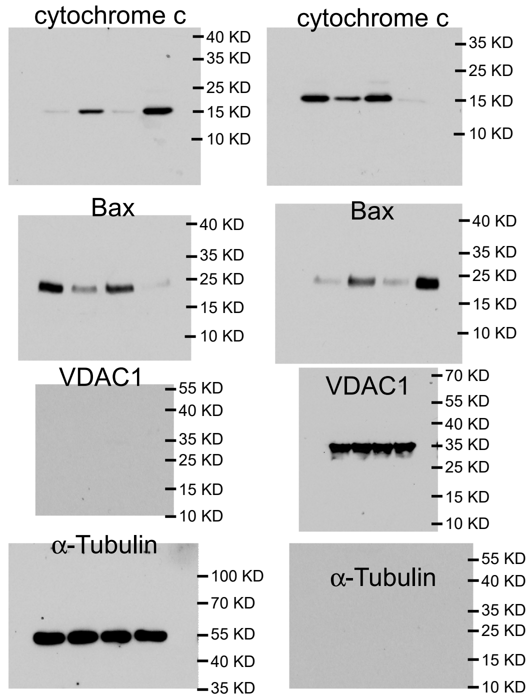

For Figure 3A

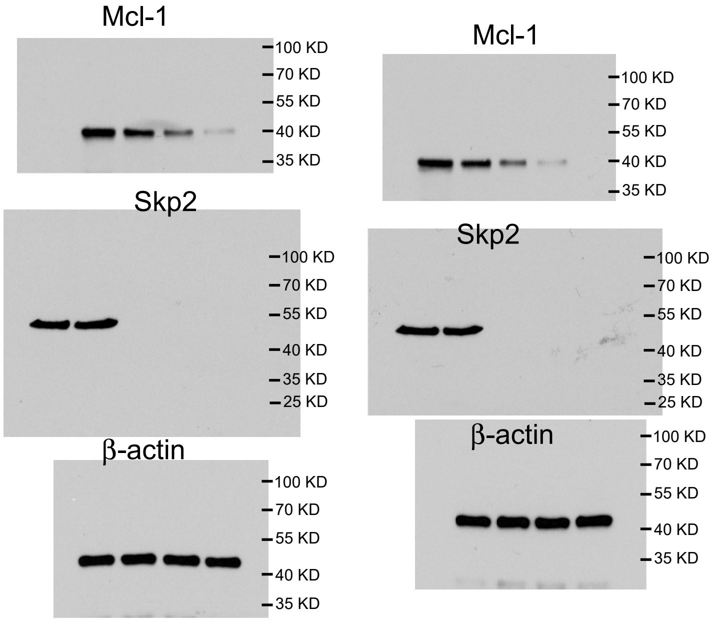

For Figure 3B

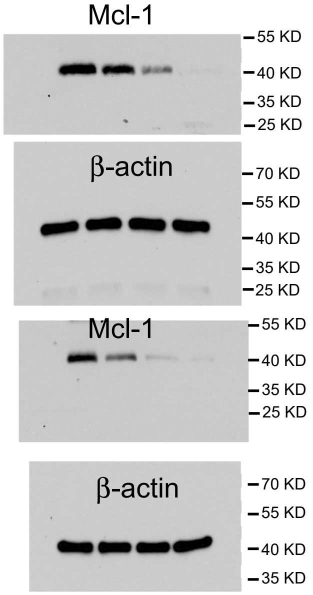

For Figure 3E

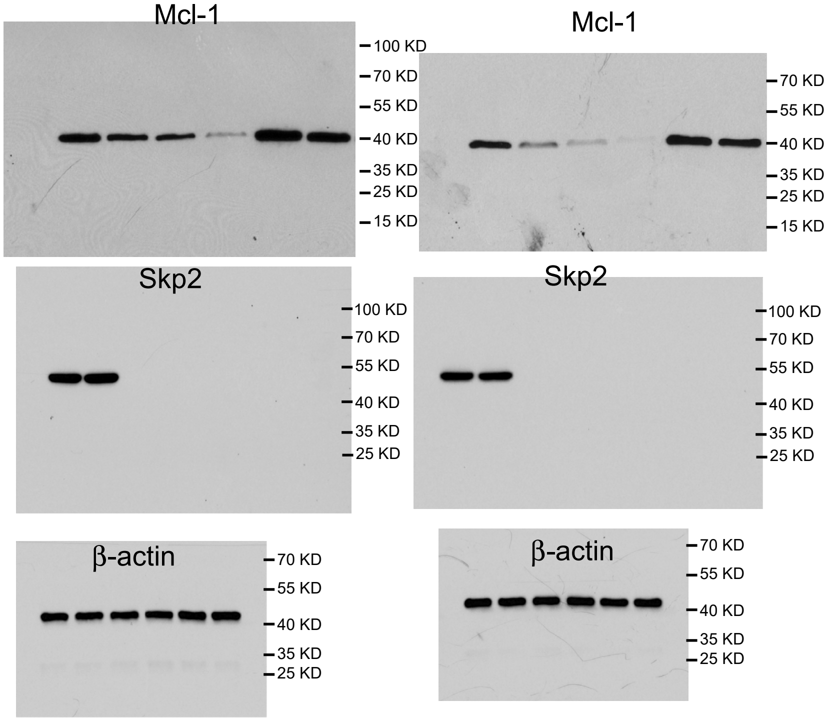

For Figure 4B

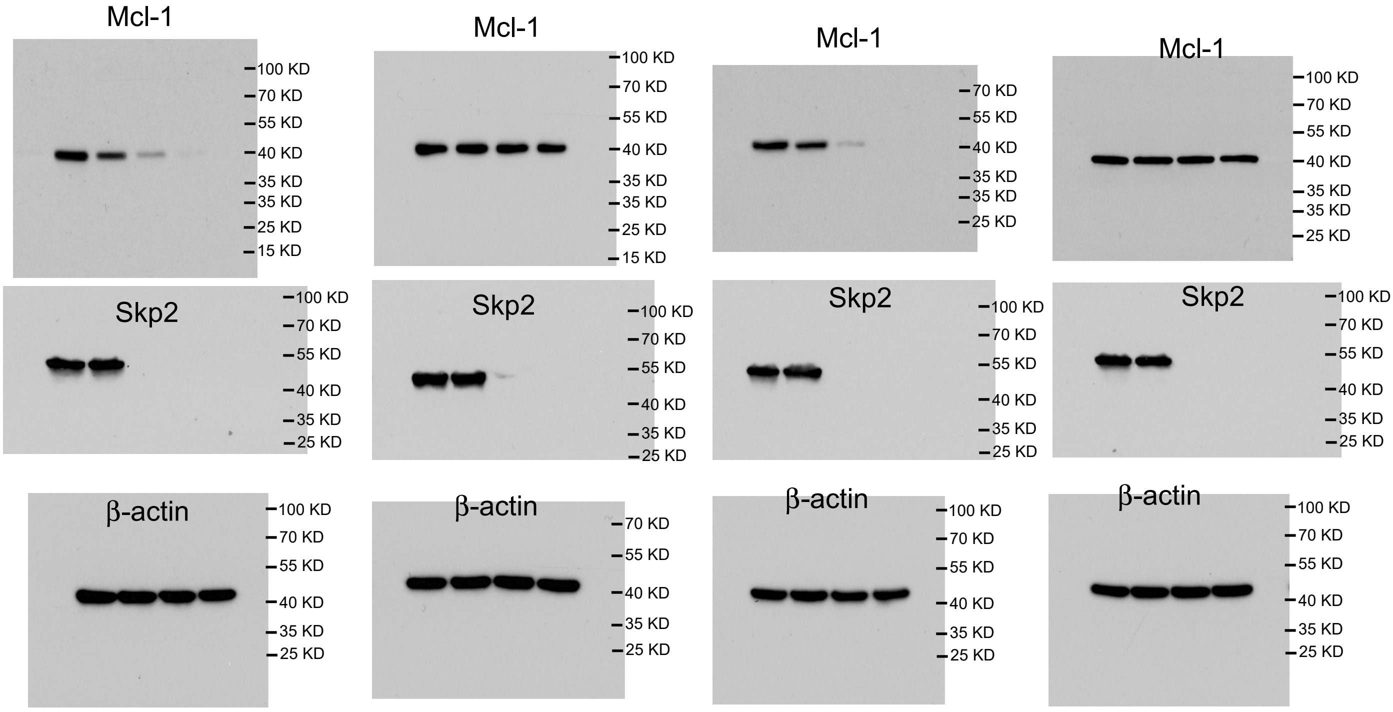

For Figure 4C

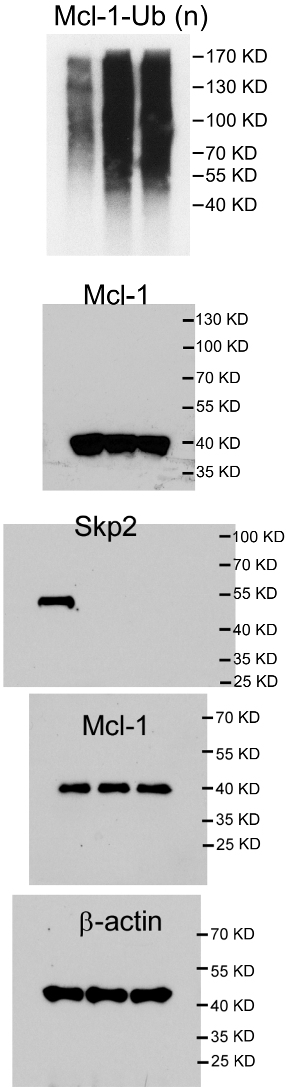

D

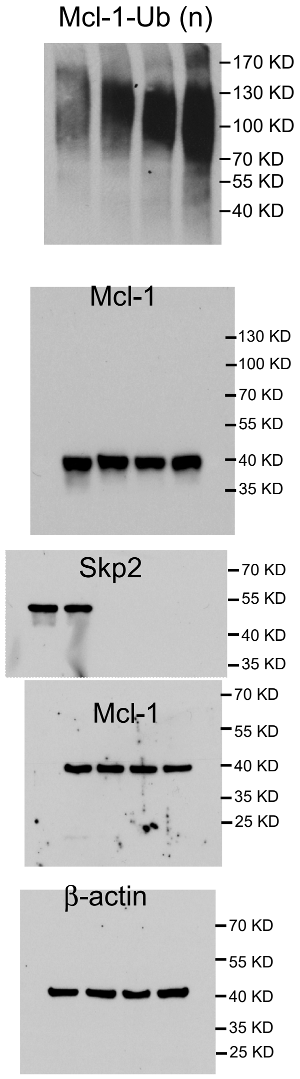

E

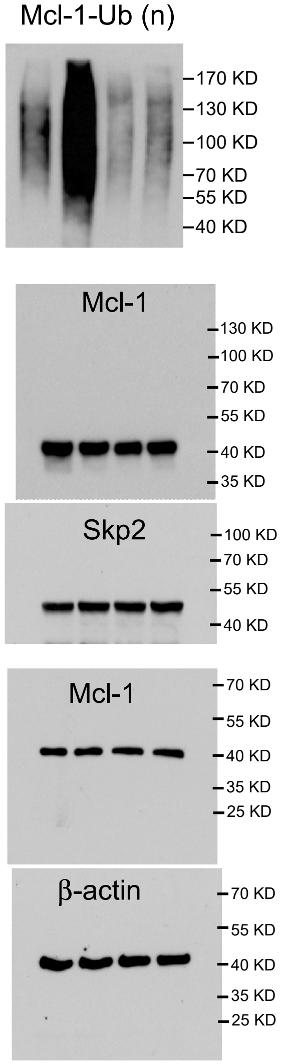

F

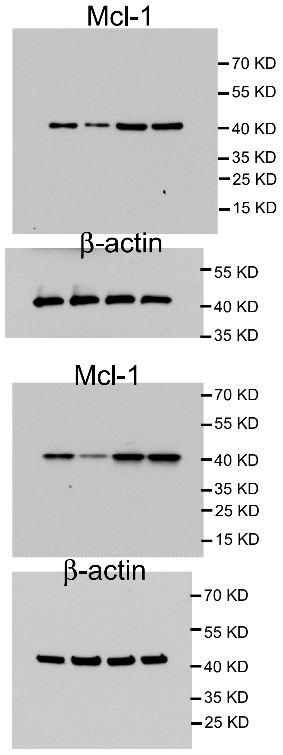

For Figure 5

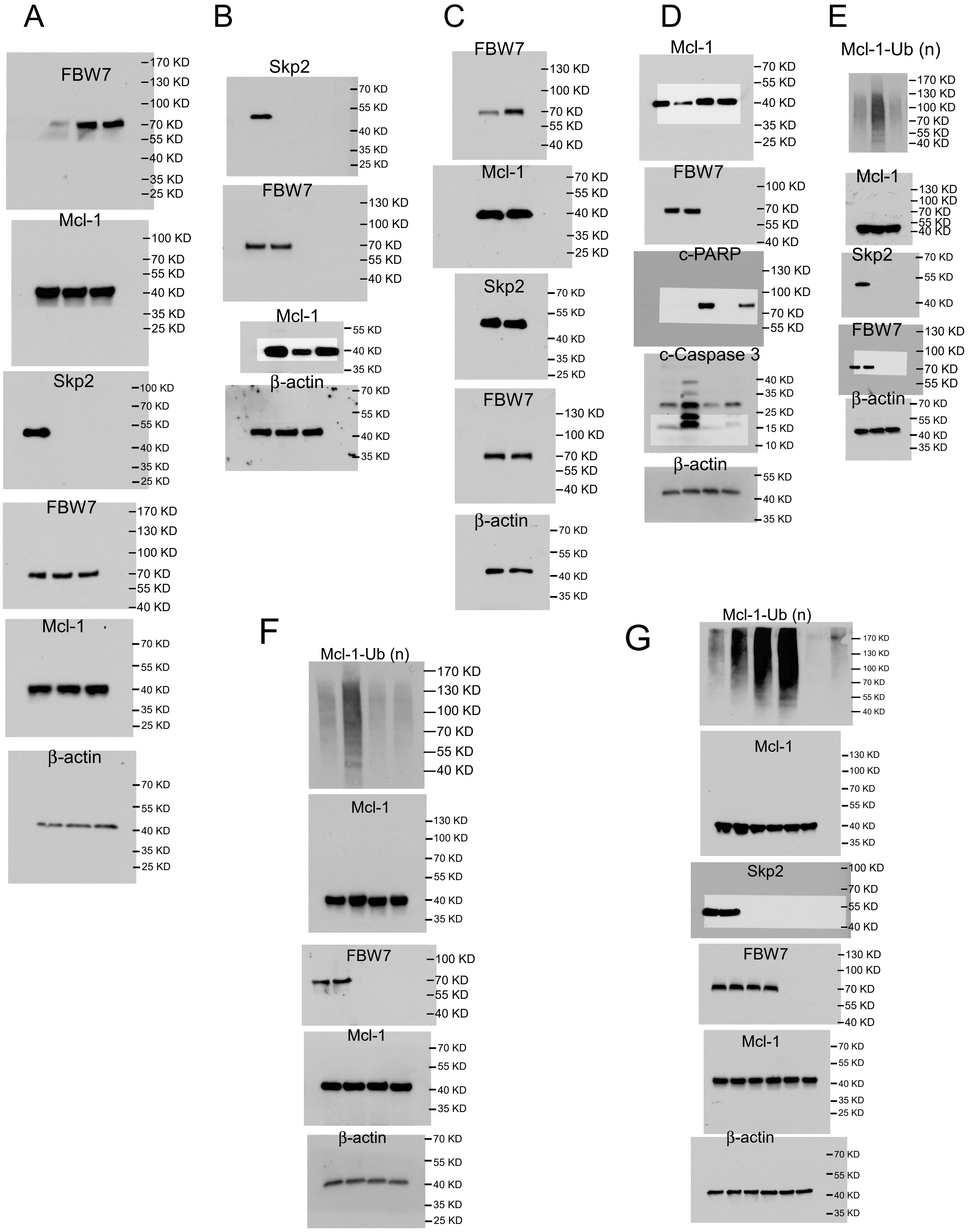
A
FBW7
170 KD
130 KD
100 KD
70 KD
55 KD
40 KD
35 KD
25 KD
Mcl-1
100 KD
70 KD
55 KD
40 KD
35 KD
25 KD
Skp2
100 KD
70 KD
55 KD
40 KD
35 KD
25 KD
FBW7
170 KD
130 KD
100 KD
70 KD
55 KD
40 KD
Mcl-1
70 KD
55 KD
40 KD
35 KD
25 KD
β-actin
70 KD
55 KD
40 KD
35 KD
25 KD
B
Skp2
70 KD
55 KD
40 KD
35 KD
25 KD
FBW7
130 KD
100 KD
70 KD
55 KD
40 KD
Mcl-1
55 KD
40 KD
35 KD
β-actin
70 KD
55 KD
40 KD
35 KD
C
FBW7
130 KD
100 KD
70 KD
55 KD
40 KD
Mcl-1
70 KD
55 KD
40 KD
35 KD
25 KD
Skp2
70 KD
55 KD
40 KD
35 KD
25 KD
FBW7
130 KD
100 KD
70 KD
55 KD
40 KD
β-actin
70 KD
55 KD
40 KD
35 KD
D
Mcl-1
70 KD
55 KD
40 KD
35 KD
25 KD
FBW7
100 KD
70 KD
55 KD
40 KD
c-PARP
130 KD
100 KD
70 KD
55 KD
c-Caspase 3
40 KD
35 KD
25 KD
15 KD
10 KD
β-actin
55 KD
40 KD
35 KD
E
Mcl-1-Ub (n)
170 KD
130 KD
100 KD
70 KD
55 KD
40 KD
Mcl-1
130 KD
100 KD
70 KD
55 KD
40 KD
Skp2
70 KD
55 KD
40 KD
FBW7
130 KD
100 KD
70 KD
55 KD
β-actin
70 KD
55 KD
40 KD
35 KD
F
Mcl-1-Ub (n)
170 KD
130 KD
100 KD
70 KD
55 KD
40 KD
Mcl-1
130 KD
100 KD
70 KD
55 KD
40 KD
35 KD
FBW7
100 KD
70 KD
55 KD
40 KD
Mcl-1
70 KD
55 KD
40 KD
35 KD
β-actin
70 KD
55 KD
40 KD
35 KD
25 KD
G
Mcl-1-Ub (n)
170 KD
130 KD
100 KD
70 KD
55 KD
40 KD
Mcl-1
130 KD
100 KD
70 KD
55 KD
40 KD
35 KD
Skp2
100 KD
70 KD
55 KD
40 KD
FBW7
130 KD
100 KD
70 KD
55 KD
40 KD
Mcl-1
70 KD
55 KD
40 KD
35 KD
25 KD
β-actin
70 KD
55 KD
40 KD
35 KD

For Figure S1

A

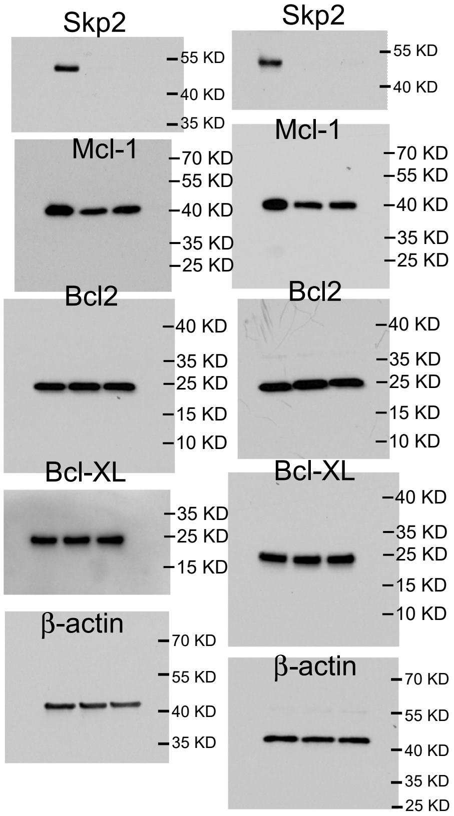

D

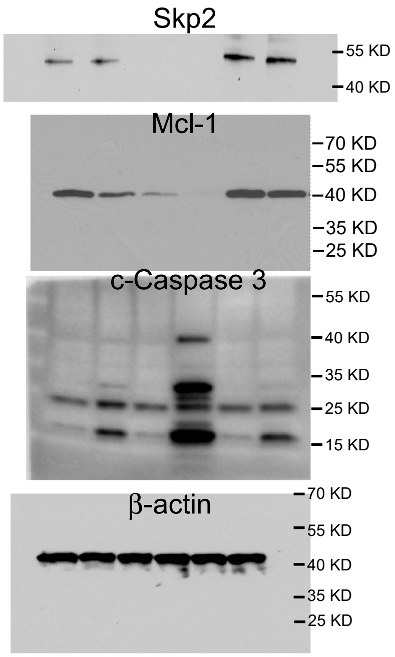

For Figure S2

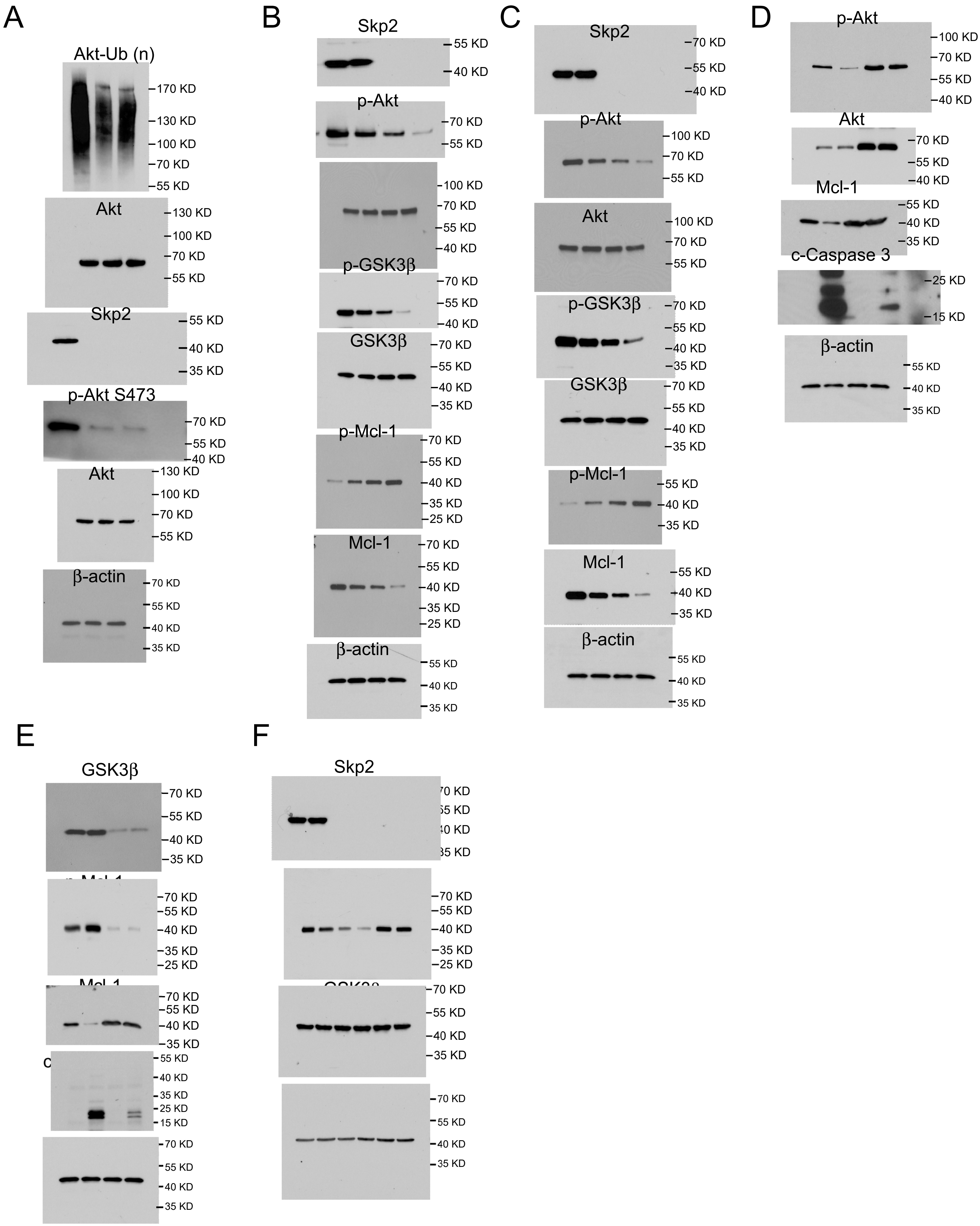

For Figure S3

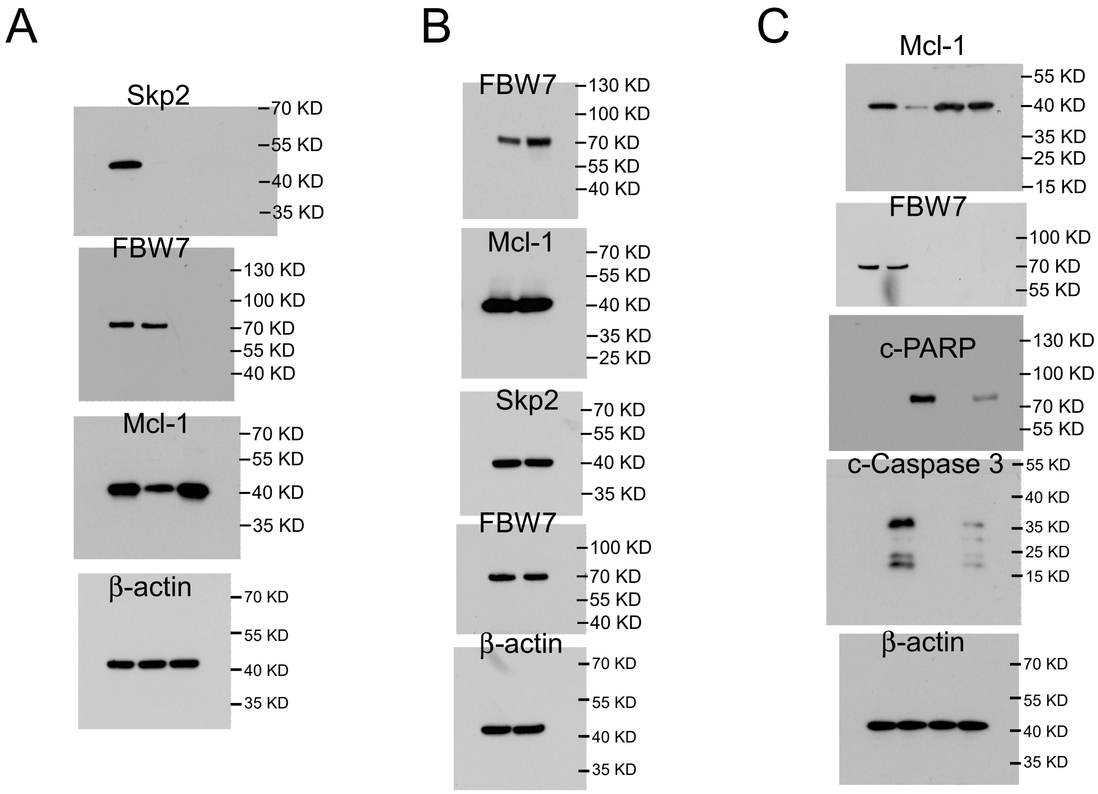

Supplement: Supplementary file 1 — Combined Supplementary Information [file 41419_2022_4685_MOESM1_ESM.pdf]
